# Anticholinesterase activities of novel isoindolin-1,3-dione-based acetohydrazide derivatives: design, synthesis, biological evaluation, molecular dynamic study

**DOI:** 10.1186/s13065-024-01169-4

**Published:** 2024-04-01

**Authors:** Ahmad Nazarian, Fahime Abedinifar, Haleh Hamedifar, Mohammad Hashem Hashempur, Mohammad Mahdavi, Nima Sepehri, Aida Iraji

**Affiliations:** 1 Endocrinology and Metabolism Research Center, Endocrinology and Metabolism Clinical Sciences Institute, Tehran University of Medical Sciences, Tehran, Iran; 2 CinnaGen Medical Biotechnology Research Center, Alborz University of Medical Sciences, Karaj, Iran; 3 CinnaGen Research and Production Co., Alborz, Iran; 4 Research Center for Traditional Medicine and History of Medicine, Department of Persian Medicine, School of Medicine, Shiraz University of Medical Sciences, Shiraz, Iran; 5 Stem Cells Technology Research Center, Shiraz University of Medical Sciences, Shiraz, Iran

**Keywords:** Acetylcholinesterase, Butyrylcholinesterase, Isoindolin-1,3-dione, Molecular dynamics simulations

## Abstract

In pursuit of developing novel cholinesterase (ChE) inhibitors through molecular hybridization theory, a novel series of isoindolin-1,3-dione-based acetohydrazides (compounds **8a**–**h**) was designed, synthesized, and evaluated as possible acetylcholinesterase (AChE) and butyrylcholinesterase (BChE) inhibitors. In vitro results revealed IC_50_ values ranging from 0.11 ± 0.05 to 0.86 ± 0.02 µM against AChE and 5.7 ± 0.2 to 30.2 ± 2.8 µM against BChE. A kinetic study was conducted on the most potent compound, **8a**, to ascertain its mode of inhibition, revealing its competitive mode against AChE. Furthermore, the binding interaction modes of the most active compound within the AChE active site was elucidated. Molecular dynamics simulations of compound **8a** were performed to assess the stability of the **8a**-AChE complex. In silico pharmacokinetic predictions for the most potent compounds indicated their potential as promising lead structure for the development of new anti-Alzheimer’s disease (anti-AD) agents.

**Supplementary Information:**

The online version contains supplementary material available at 10.1186/s13065-024-01169-4.

## Introduction

Alzheimer’s disease (AD) is a multifaceted neurodegenerative condition affecting a substantial portion of the global population, with an increasing prevalence as life expectancy rises. AD poses significant challenges not only to individuals and their families but also exacts a considerable economic burden on society. The escalating number of AD cases places an unprecedented burden on healthcare systems and social services, resulting in substantial financial costs [1, 2].

The pathogenesis of AD is complex and involves various molecular, cellular, and neurochemical changes. One significant aspect of AD pathophysiology is cholinergic system dysfunction, particularly the deficiency of acetylcholine (ACh), a neurotransmitter crucial for cognitive functions. Cholinergic neurons progressively degenerate in AD, substantially reducing ACh levels in critical brain regions associated with learning and memory [3]. This cholinergic deficit is exacerbated by acetylcholinesterase (AChE) activity, which diminishes ACh availability. The resulting disruption in cholinergic neurotransmission contributes significantly to the cognitive decline observed in AD, emphasizing the importance of addressing cholinergic dysfunction in therapeutic interventions [4, 5].

Crystallographic investigations of AChE demonestrated two distinct binding sites. The primary site is situated at the base of a deep and narrow 20 Å gorge, encompassing the AChE catalytic triad and an anionic subsite called the catalytic active site (CAS). Additionally, there exists the peripheral anionic site (PAS) located near the entrance of the gorge [6]. The PAS plays a pivotal role, as it is thought to facilitate the aggregation process of amyloid-beta (Aβ), contributing to the intricate molecular mechanisms associated with AD [7].

Butyrylcholinesterase (BChE) is another enzyme related to cholinergic neurotransmission, and it plays a role similar to AChE in breaking cholinergic neurotransmitters. While AChE is more abundant in the central nervous system, BChE is found in various tissues throughout the body and the brain [8]. In the context of AD, the primary focus has been done on AChE due to its prominent role in the cholinergic system [9, 10]. However, researchers have also investigated the involvement of BChE in AD pathology [10]. Current AD therapies primarily revolve around the cholinergic hypothesis and involve the use of cholinesterase inhibitors, including medications such as donepezil, galantamine, and rivastigmine. Ongoing research aims to explore novel AChE and BChE inhibitors that not only alleviate symptoms but also potentially modify the course of the disease, offering hope for more effective interventions in the future, and different scaffolds have been developed as effective anti-ChE inhibitors,including diazepine [11], hydrazone–sulfonate [12], benzimidazole [13], isatin [14], benzoxazole [14], imidazole [15] derivatives.

Isoindolin-1,3-dione, a bicyclic *N*-heterocycle, has demonstrated diverse biological effects, including antimicrobial activity [15], histone deacetylase inhibition [16], EGFR-TK inhibition [17], and analgesic activity [18]. In recent years, several series of synthetic isoindolin-1,3-dione derivatives with notable anti-ChE activity have been reported [19]. Among these, derivative **A**, Table 1 showcased a potent compound inhibiting human AChE with an IC_50_ of 0.361 μM [20]. Another compound, 2-(benzylamino-2-hydroxyalkyl)isoindoline-1,3-dione (**B**, Table 1), demonstrated significant inhibitory activity against both AChE and BACE1. Molecular docking studies revealed interactions with the PAS and CAS [21]. Remarkably, longer linkers were found to be more favorable for interactions with the PAS and CAS pocket [22].

**Table 1:** Structure and potency of previously reported anti-AD candidates

Comp	Structure	Activity
**A**		hAChE IC_50_ = 0.361 µM vs donepezil with IC_50_ = 0.006 ± 0.001 µM hBChE IC_50_ > 1000 µM vs donepezil with IC_50_ = 1.830 ± 0.176 µM PAMPA-BBB = + vs progesterone with PAMPA-BBB = +
**B**		AChE IC_50_ = 3.33 µM vs donepezil with IC_50_ = 0.011 ± 0.0002 µM BChE = 14.1% ± 5.8 at 10 µM vs donepezil with IC_50_ = 1.83 ± 0.04 µM hBACE-1 = 43.7% inhibition at 50 µM vs calbiochem with IC_50_ = 0.046, Aß-aggregation = 24.9% inhibition at 10 µM vs resveratrol with 78.5% ± 5.2 inhibition
**c**		AChE IC_50_ = 53.1 ± 4.56 µM vs donepezil with IC_50_ = 23 nM BChE IC_50_ = 67.3 ± 5.24 µM vs donepezil with IC_50_ = 7.4 µM
**D**		AChE IC_50_ = 0.0465 µM vs neostigmine with IC_50_ = 0.136 µM BChE IC_50_ = 0.0702 µM vs neostigmine with IC_50_ = 0.084 µM

Expanding on the foundation provided by donepezil as a lead structure for AChE inhibition, a series of potent derivatives featuring a benzylamine moiety were precisely developed. The incorporation of benzylamine into the acetohydrazide group gave rise to a novel ChE inhibitor, designated as compound **C**, Table 1. In-depth in silico studies underscored critical interactions with both the PAS and CAS, underscoring the pivotal role of the acetohydrazide linker in the compound's inhibitory activity [23]. Moreover, this acetohydrazide linker has been prominently featured in various investigations. Its presence in potent ChE inhibitors is exemplified by compound **D**, Table 1, demonstrated a remarkable increase in potency compared to the positive control, neostigmine [24]. These findings collectively highlight the strategic significance of the acetohydrazide linker in the design and development of potent ChE inhibitors, shedding light on potential avenues for designing more effective therapeutic agents.

**Fig. 1:**
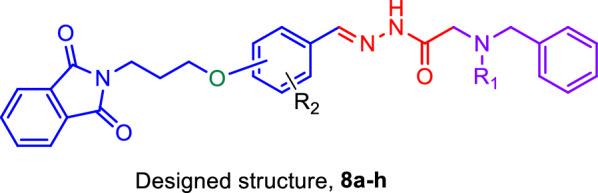
Design strategy for the new ChE inhibitors **8a**–**h**

In the design strategy for new ChE inhibitors, novel molecules with an isoindolin-1,3-dione heterocyclic scaffold linked to acetohydrazidestructures were synthesized (Fig. 1). These molecules were evaluated for their potential as anti-AD agents through in vitro AChE and BChE inhibition assays. Kinetic studies of the most potent derivative was performed, and to gain further insights into their interaction with ChE, molecular docking and molecular dynamics simulation studies were conducted. This comprehensive approach contributes to the ongoing efforts to develop advanced therapeutics for AD.

## Result and discussion

### Chemistry

**Scheme 1:**
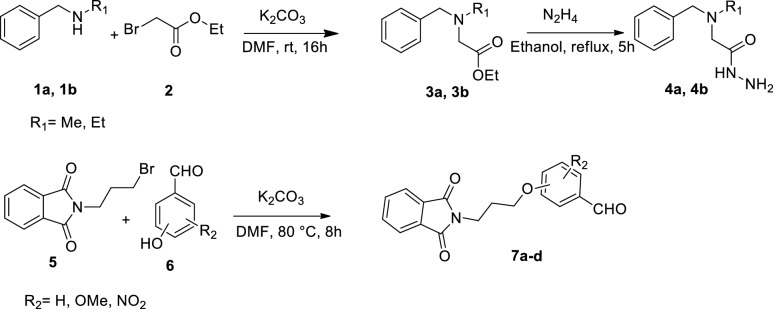
Synthetic procedure of **7a**–**d**

The synthetic pathway for the production of isoindolin-1,3-dione-based compounds is illustrated in Scheme 1. Initially, a secondary amines *N*-methyl-1-phenylmethanamine (**1a)** or *N*-ethyl-1-phenylmethanamine (**1b**) solution containing potassium carbonate (K_2_CO_3_) in DMF (20 mL) was prepared, and ethyl bromoacetate (**2**) was added. The reaction mixture was stirred at room temperature for 16 h to yield derivative **3**. Subsequently, compound **3 **was dissolved in ethanol, and hydrazine hydrate was added to the solution. The mixture was refluxed for one hour, followed by adding an additional amount of hydrazine hydrate. The reaction mixture was refluxed for an additional 4 h, forming product **4** as a colorless oil in good yields. Next, 2-(3-bromopropyl)isoindoline-1,3-dione (compound **5**) was reacted with substituted hydroxybenzaldehyde (**6**) in the presence of potassium carbonate in DMF, yielding intermediate** 7**.

**Scheme 2:**
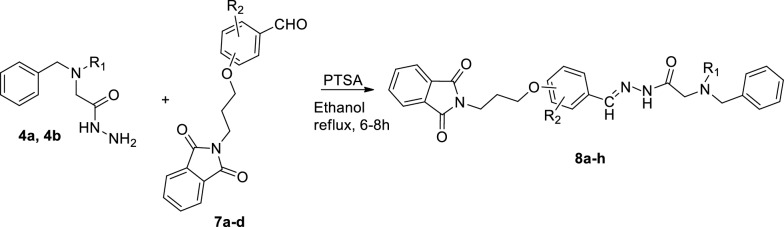
Synthetic procedure of **8a**–**h**

Finally, to a solution of **4** containing a catalytic amount of *p*-Toluenesulfonic acid (*p*-TSA) in ethanol, intermediate **7a**–**d** was added. The reaction mixture was refluxed for 8 h, and the solid product was filtered, washed with ethanol, and dried under reduced pressure to yield the final products, **8a**–**h** (Scheme 2).

### Biological studies

This study determined the inhibitory effects of the compounds **8a**–**h** againstAChE and the enzymes compared with donepezil as a positive control in terms of IC_50_ (Table 2).

**Table 2:** AChE and BChE inhibitory activities of analogs **8a**–**h** 
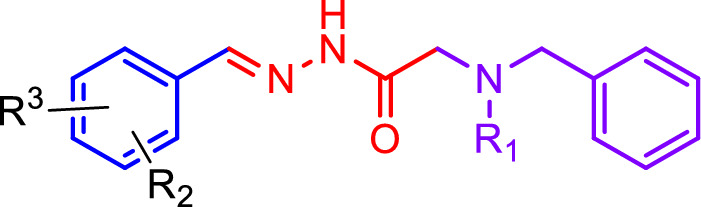

Comp	R^1^	R^2^	R^3^	AChE IC_50_ (µM)	BChE IC_50_ (µM)
8a	Methyl	H	2-oxypropyl-isoindolinedione	0.11 ± 0.05	30.2 ± 2.8
8b	Ethyl	H	2-oxypropyl-isoindolinedione	0.18 ± 0.01	26.8 ± 0.8
8c	Ethyl	5-NO_2_	2-oxypropyl-isoindolinedione	0.16 ± 0.04	21.8 ± 1.3
8d	Ethyl	H	3-oxypropyl-isoindolinedione	0.16 ± 0.03	11.8 ± 0.5
8e	Ethyl	4-OCH_3_	3-oxypropyl-isoindolinedione	0.21 ± 0.03	14.7 ± 0.2
8f	Methyl	H	4-oxypropyl-isoindolinedione	0.32 ± 0.02	15.7 ± 1.2
8g	Ethyl	H	4-oxypropyl-isoindolinedione	0.86 ± 0.02	5.7 ± 0.2
8h	Ethyl	3-OCH_3_	4-oxypropyl-isoindolinedione	0.17 ± 0.06	13.2 ± 1.4
Donepezil^a^	–	–	–	0.023 ± 0.02	7.2 ± 0.1

IC_50_ values are indicated as the mean ± SD

^a^Positive control

Compound **8a**, featuring a methyl group at R^1^ and 2-oxypropyl-isoindolinedionemoiety at R^3^, exhibited remarkable potency against AChE, boasting an IC_50_ value of 0.11 µM. Conversely, this derivative displayed weaker activity against BChE, with an IC_50_ value of 30.2 µM. To determine the impact of substitution at the R^1^ position on inhibitory activity, compound **8b**, incorporating an ethyl group at the R^1^ position, was synthesized. Interestingly, a marginal reduction in AChE potency and a slight improvement in BChE activity were observed. This phenomenon correlated with the size disparity between the active sites of AChE and BChE, wherein BChE possesses a larger active site. Subsequently, a modification was done wherein a 5-NO_2_ (electron-withdrawing and conducive to hydrogen bonding interactions) group was substituted at the R^2^ position, resulting in compound **8c**. An enhancement in potency against both AChE and BChE was evident compared to **8b**. This highlights the significance of strategic molecular modifications in tailoring compound interactions with specific enzyme targets.

Next, derivatives **8d** and **8e** were synthesized, wherein the oxypropyl-isoindolinedione group at R^3^ was shifted to the *meta* position. Upon initial comparison between **8d** and **8b**, a slight improvement in AChE inhibition was observed, accompanied by a notable enhancement in BChE inhibition. This validates that the *meta* position is more favorable than the *ortho* position for this group. However, introducing a 4-OCH_3_ serving as a strong electron-donating moiety at the R^2^ position in **8e** resulted in reduced potency compared to **8d**. This outcome underscores that such a modification is unfavorable to optimal inhibitory activity.

Furthermore, derivatives **8f**–**h** bearing 4-oxypropyl-isoindolinedione group at R^3^ were synthesized. Compound **8f**, featuring a methyl group at R^1^ and oxypropyl-isoindolinedione group in the *para* position of R^3^, demonstrated a reduction in AChE inhibition with an IC_50_ value of 0.32 ± 0.02 µM. Conversely, the inhibitory potency deteriorated in **8g**, where an ethyl group was substituted at the R^1^ position, resulting in an IC_50_ value of 0.86 ± 0.02 µM.

In the context of AChE inhibition, the most active analogs was **8a** (IC_50_ = 0.11 ± 0.05 µM against AChE and IC_50_ = 30.2 ± 2.8 µM against BChE) compared with donepezil as positive control with IC_50_ = 0.023 ± 0.02 µM against AChE and IC_50_ = 7.2 ± 0.1 µM against BChE. It is apparent that a smaller and less elongated molecule is more favorable for interaction with the active site. Consequently, *ortho* substitution with the oxypropyl-isoindolinedione group proves to be the most advantageous, followed by *meta* and *para* positions. An exception to this trend is observed in compound **8h**, which bears a 3-OCH_3_ substitution at the R^2^ position. Furthermore, a smaller substituent, such as methyl at R^1^, demonstrates greater efficacy compared to ethyl for AChE inhibition.

Notably, an interesting and important reversal of trends is observed in BChE inhibition. Compound **8g** is categorized as the most potent BChE inhibitor, exhibiting an IC_50_ value of 5.7 ± 0.2 µM and IC_50_ = 0.86 ± 0.02 µM against AChE, followed by **8d** (IC_50_ = 0.16 ± 0.03 µM against AChE and IC_50_ = 11.8 ± 0.5 µM against BChE) and **8h** (IC_50_ = 0.17 ± 0.06 µM against AChE and IC_50_ = 13.2 ± 1.4 µM against BChE). Donepezil as positive control exhibited IC_50_ = 0.023 ± 0.02 µM against AChE and IC_50_ = 7.2 ± 0.1 µM against BChE. This underscores that the presence of oxypropyl-isoindolinedione at the *para* position of R^3^ is particularly favorable for BChE inhibition. These results emphasize that an elongated and spacious molecular structure is more conducive to effectively inhibiting BChE. The structural insights gained from this study can contribute to the rational design of compounds with enhanced BChE inhibitory activity for potential therapeutic applications.

### Kinetic study

**Fig. 2:**
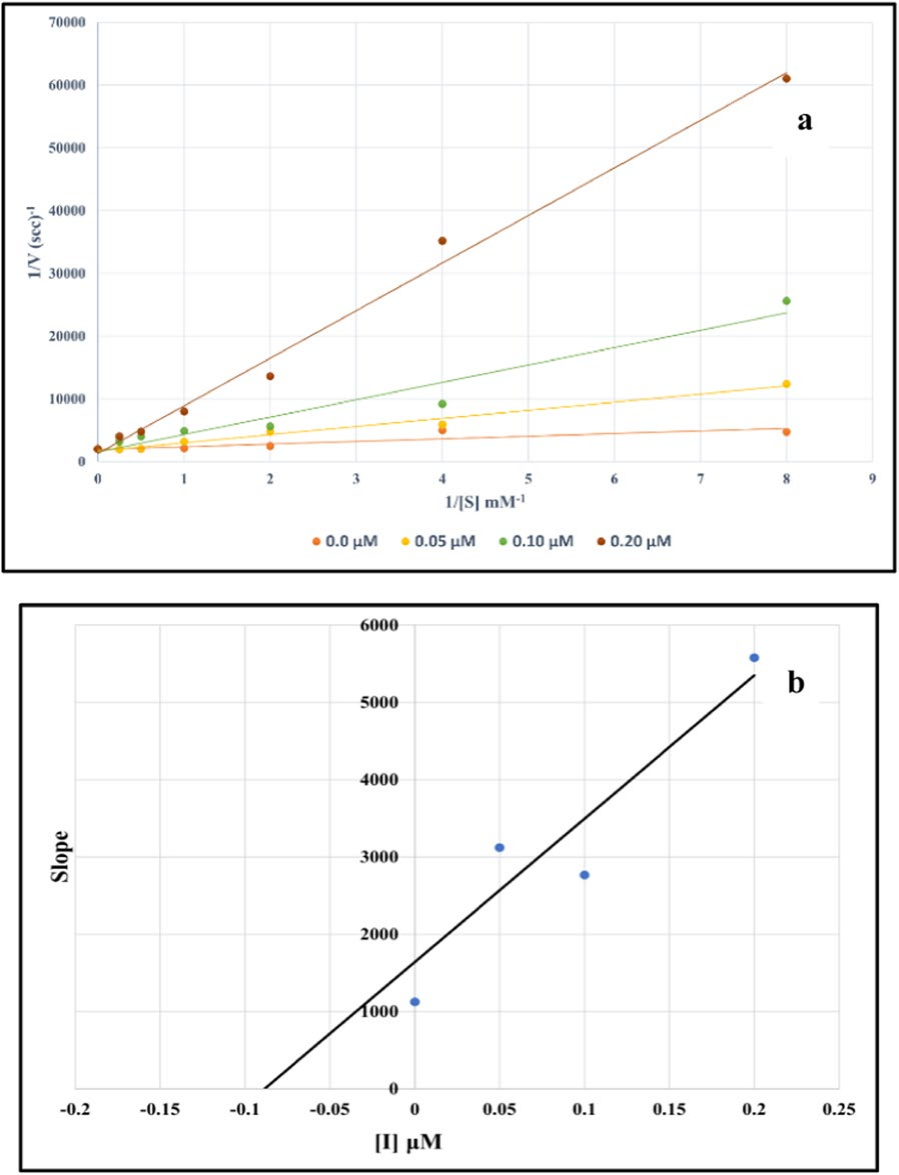
Inhibitory kinetics of compound **8a** against AChE. **a** Lineweaver–Burk plots for inhibition of compound **8a**. **b** The secondary plot of Lineweaver–Burk plots for determination *K*_*i*_ value of compound **8a**

A kinetic study was performed on the most active compound **8a,** to evaluate the inhibition mechanism. As shown in Fig. 2a, the lines of the Lineweaver–Burk plot with enhancement in the concentration of inhibitor **8a** had a fixed intercept on the Y-intercept and X-slopes. Therefore, values of *V*_*max*_ remained constant while the values of *K*_*m*_ increased. The obtained data showed that compound **8a** was a competitive inhibitor (Fig. 2a).

Furthermore, the *K*_*i*_ value, representing the inhibition constant, was determined to be 0.0886 µM. This value was obtained through a secondary plot of Lineweaver–Burk plots, as illustrated in Fig. 2b. The competitive nature of the inhibition and the low *K*_*i*_value further emphasize the affinity and effectiveness of compound **8a** in selectively interfering with the enzymatic activity, providing valuable insights into its inhibitory mechanism.

**Fig. 3:**
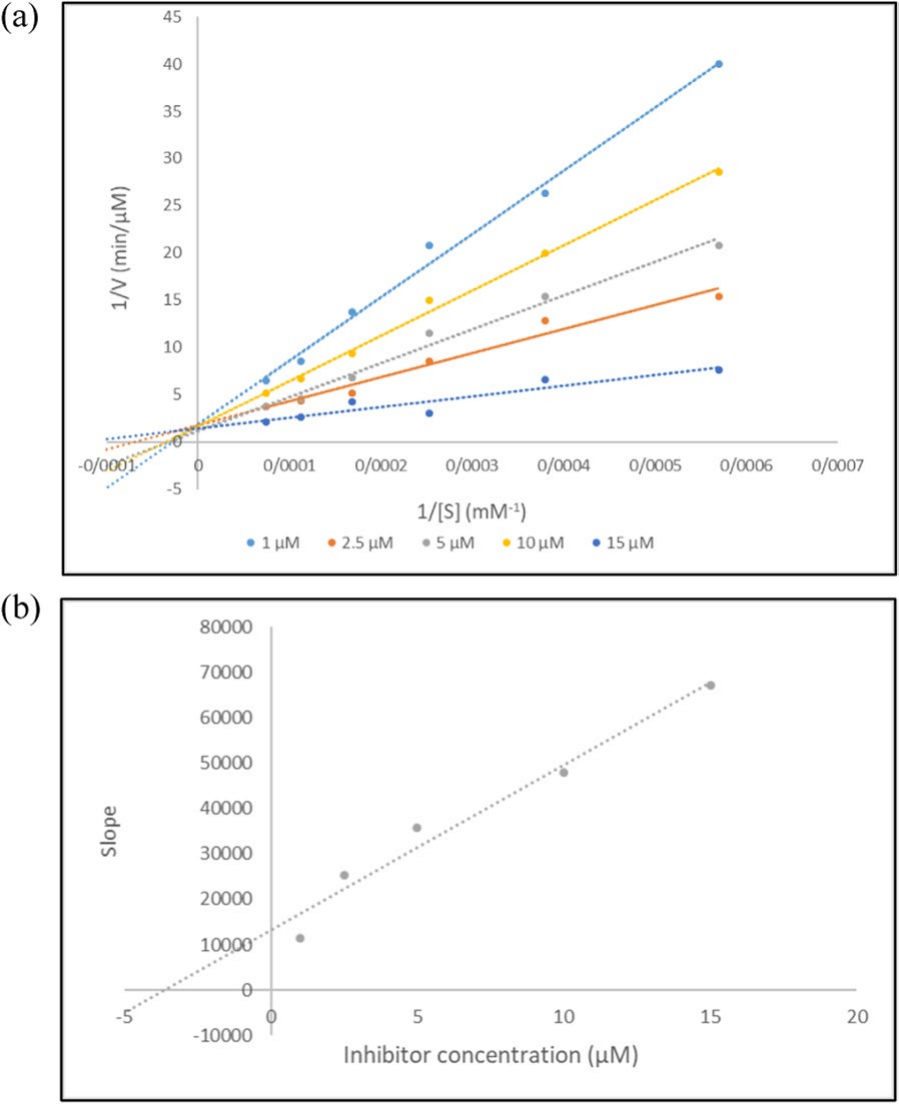
Inhibitory kinetics of compound **8g** against BChE. **a** Lineweaver–Burk plots for inhibition of compound **8g**. **b** The secondary plot of Lineweaver–Burk plots for determination *K*_*i*_ value of compound **8g**

The kinetic behavior of **8g** as the most potent inhibitor against BChE was investigated. Analysis of the Lineweaver–Burk plot (see Fig. 3a) revealed a competitive mode of inhibition. Additionally, the inhibition constant (*K*_*i*_ value) was determined to be 3.65 µM, indicating the strength of the inhibition.

### Docking study

To elucidate the interactions and rationalize the activity, derivatives **8a** and **8g**, identified as potent AChE and BChE inhibitors, respectively, underwent a molecular docking study. The initial step involved molecular docking validation, demonstrating an RMSD value of less than 2 Å.

**Fig. 4:**
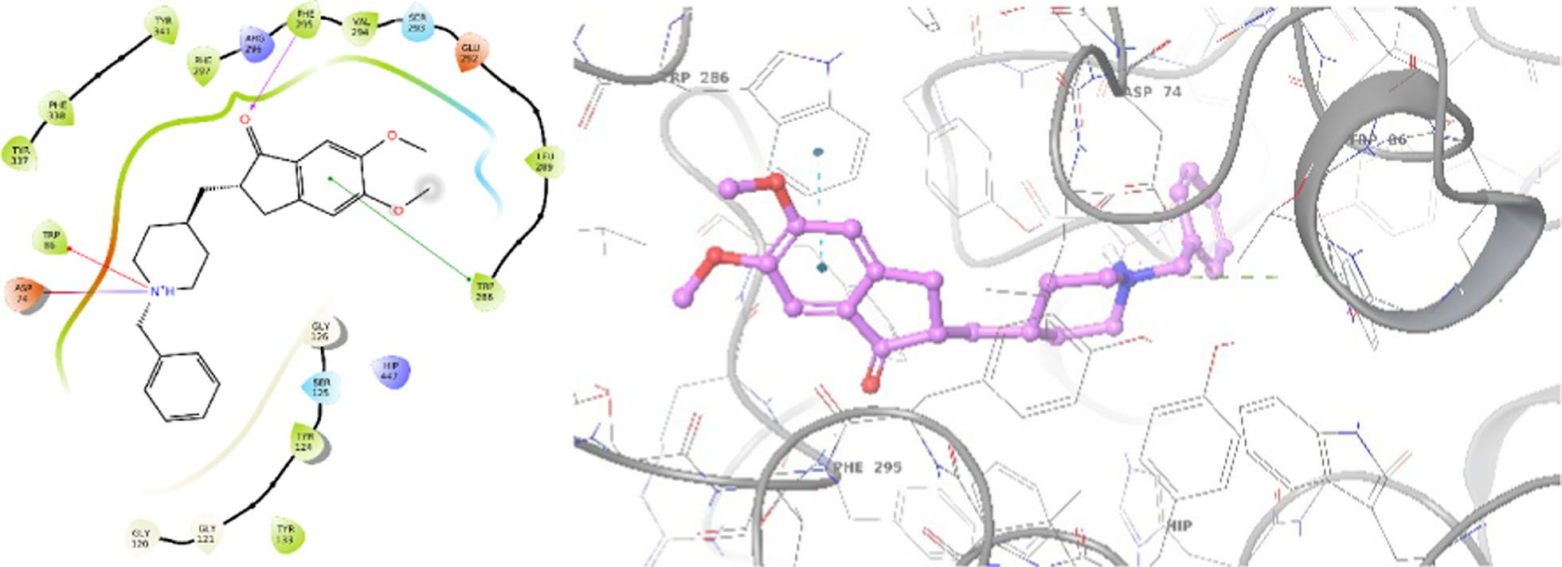
2D and 3D interaction modes of donepezil in the AChE active site

First, the interaction of donepezil as a positive control in the AChE active site was assessed. The results, illustrated in Fig. 4, indicated a binding energy of − 11.189. The carbonyl group (C=O) of inden-1-one participated in a hydrogen bond interaction with Phe295, while inden-1-one exhibited pi-pi stacking interaction with Trp286. Additionally, the benzylpiperidine tail formed a salt bridge and pi-cation interaction with Asp74 and Trp86, respectively.

**Fig. 5:**
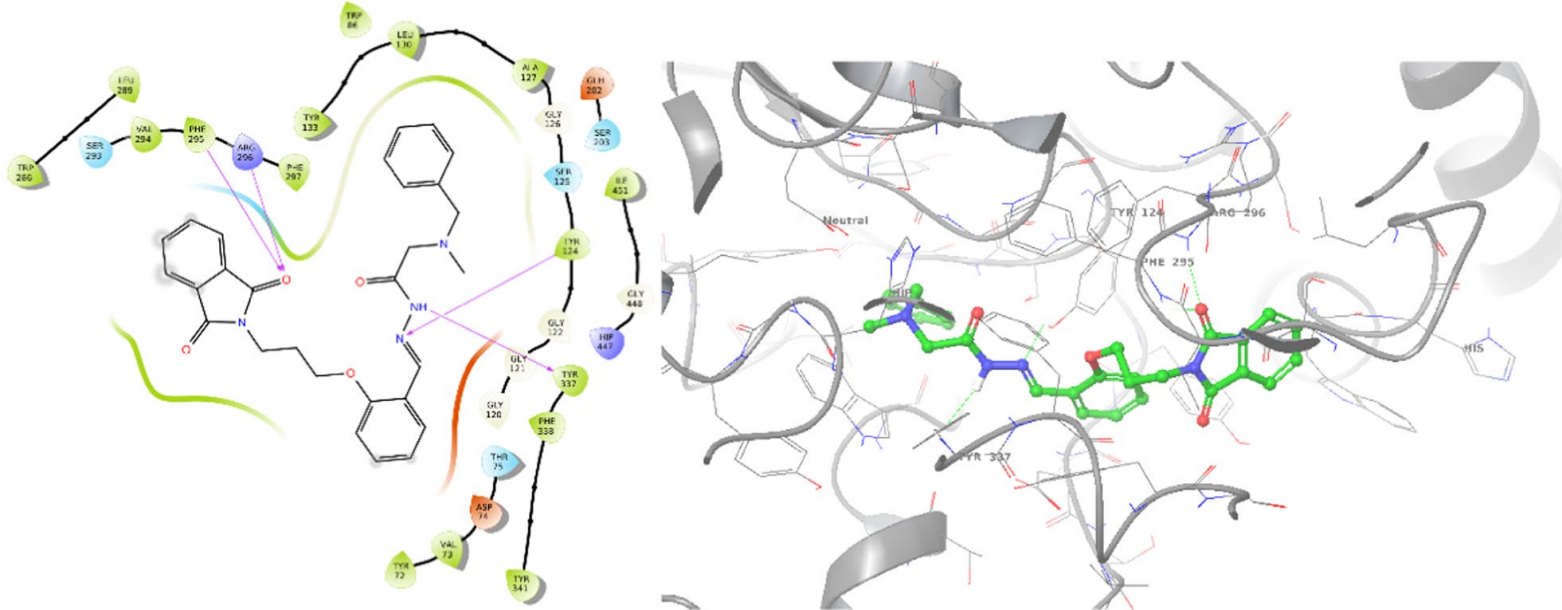
2D and 3D interaction modes of **8a** in the AChE active site

The interaction modes of **8a** are illustrated in Fig. 5, revealing a binding energy of − 10.270 kcal/mol. This compound demonstrated four hydrogen bonds with active site residues. Specifically, the C=O group of isoindolin-1,3-dione engaged in two hydrogen-bond interactions with Arg296 and Phe295 (Acyl pocket). Furthermore, the hydrazide linker participated in two additional hydrogen-bond interactions with Tyr124 and Tyr337 (PAS pocket), providing detailed insights into the molecular interactions responsible for the inhibitory activity of **8a**.

**Fig. 6:**
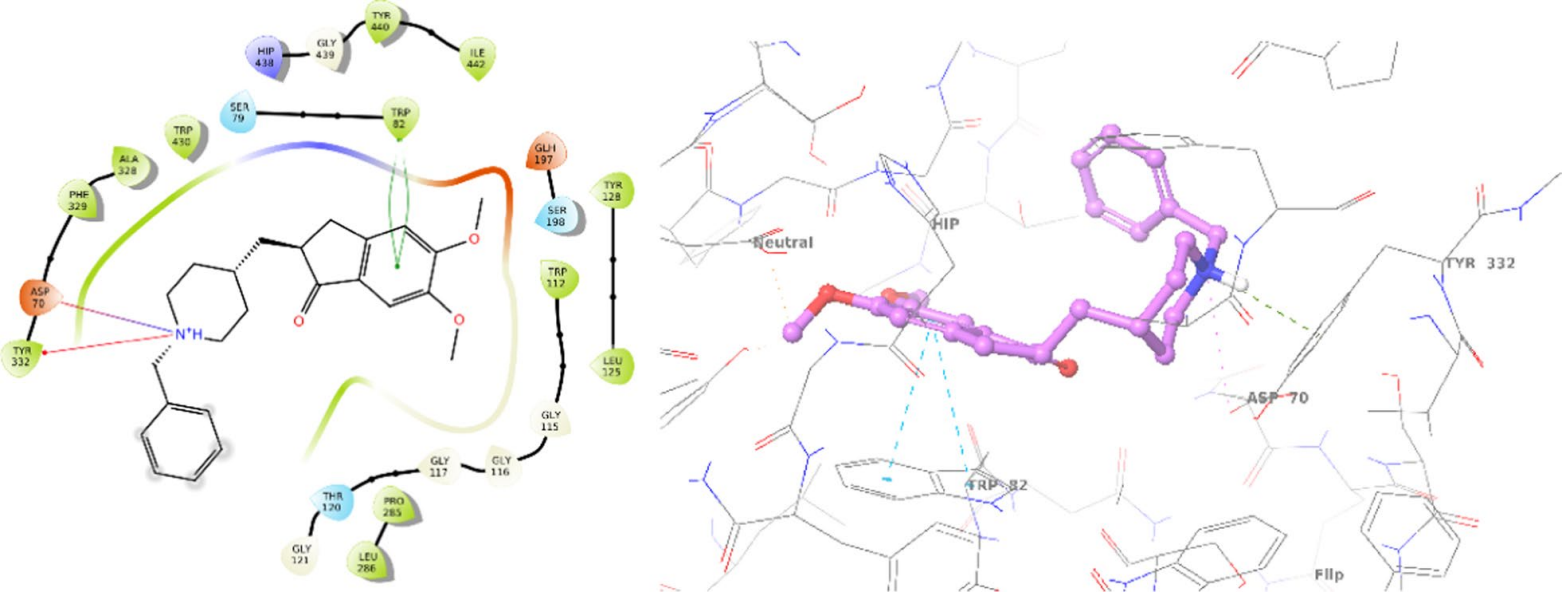
2D and 3D interaction modes of donepezil in the BChE active site

A comparative analysis was also conducted through molecular docking studies of donepezil and the most potent BChE inhibitor. Donepezil exhibited a binding energy of − 7.860 kcal/mol against BChE. As depicted in Fig. 6, inden-1-one displayed two pi-pi stacking interactions with Trp82, while the benzylpiperidine tail formed a salt bridge and pi-cation interaction with Asp70 and Trp332, respectively.

**Fig. 7:**
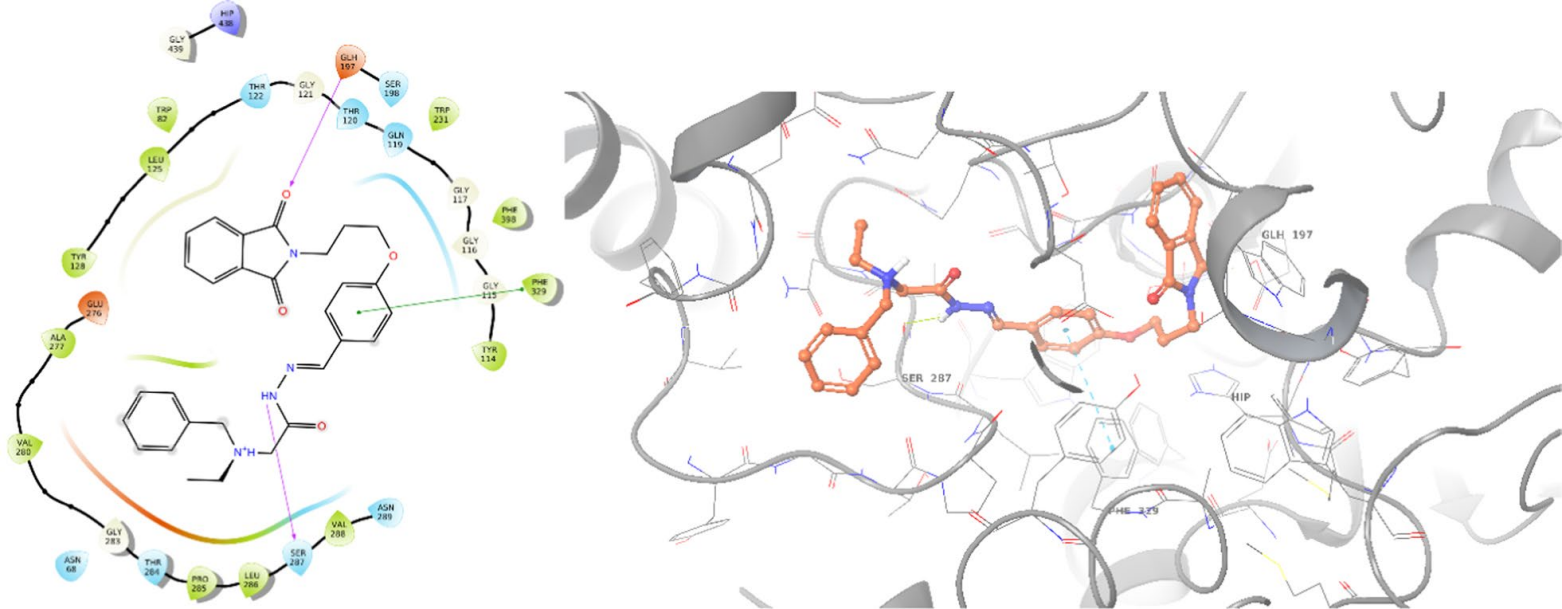
2D and 3D interaction modes of **8g** in the BChE active site

The most potent inhibitor against BChE, **8g**, exhibited a binding energy of − 9.096 kcal/mol (Fig. 7). This compound formed a hydrogen-bond interaction with Glu197 (CAS pocket) involving the C=O group of isoindolin-1,3-dione. Additionally, the phenoxy ring of **8g** established a pi–pi stacking interaction with Phe329, further enhancing its binding affinity. Moreover, the NH group of the hydrazide linker participated in another hydrogen bond interaction with Ser267. These specific molecular interactions provide insights into the structural basis of the potent inhibitory activity of compound **8g** against BChE.

### Molecular dynamic simulation

Subsequently, molecular dynamics simulation was executed for both AChE and the **8a**-AChE complex to enable a comprehensive comparison.

**Fig. 8:**
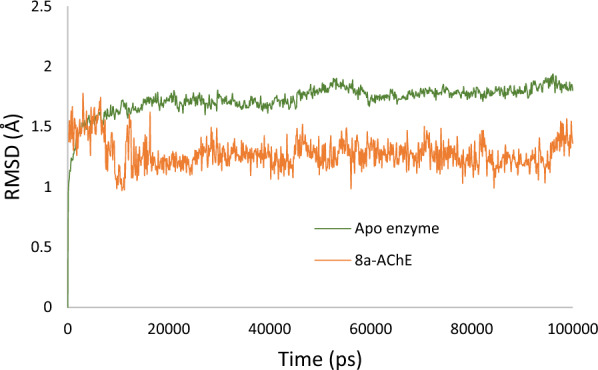
RMSD of the AChE (in green), and **8a-**AChE (in orange) for 100 ns MD simulation time

The initial step in the analysis involved evaluating the stability of compound **8a** within the AChE complex through root-mean-square deviation (RMSD) analysis. As illustrated in Fig. 8, the apoenzyme reached stability after 14 ns, with an average RMSD value of approximately ~ 1.75 Å, maintaining this stability until the end of the study. In contrast, the RMSD values for the **8a**-AChE complex exhibited a sharp increase to 1.75 Å within the initial 7 ns and then gradually decreased to 1.2 Å until the end of the study. The overall RMSD values of the **8a**-AChE system demonstrated lower values and greater stability compared to AChE alone. These findings provide compelling evidence supporting the reliability of the **8a**-enzyme optimization process, underscoring the achieved structural stability in the complex system.

**Fig. 9:**
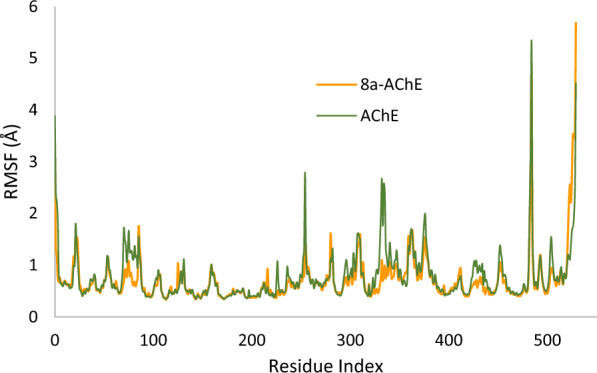
RMSF of the a-glucosidase (in green), and **8a-**AChE (in orange), for 100 ns MD simulation time

Following this, the variations in Root Mean Square Fluctuation (RMSF) were examined and compared across apo system, as depicted in Fig. 9. Notably, the apoenzyme displayed higher fluctuations throughout the simulation. Interestingly, a noticeable reduction in residue movement was observed upon the introduction of compound **8a** into the AChE binding site.

This decrease in fluctuation can be attributed to the non-bonding interactions established between the ligands and the enzyme, particularly in the regions of 66–99 (PAS), 325–346 (PAS), and 297–324, as well as 419–443, along with a notable decrease in residues 254 and 484. These findings emphasize the substantial role of the ligands in mitigating complex fluctuations, underscoring their potential significance in stabilizing the AChE-ligand complex (see Fig. 9). The reduced RMSF values suggest a more constrained and stable conformation of the AChE-ligand complex compared to the apoenzyme, supporting the positive impact of the introduced ligand on the overall stability of the system.

**Fig. 10:**
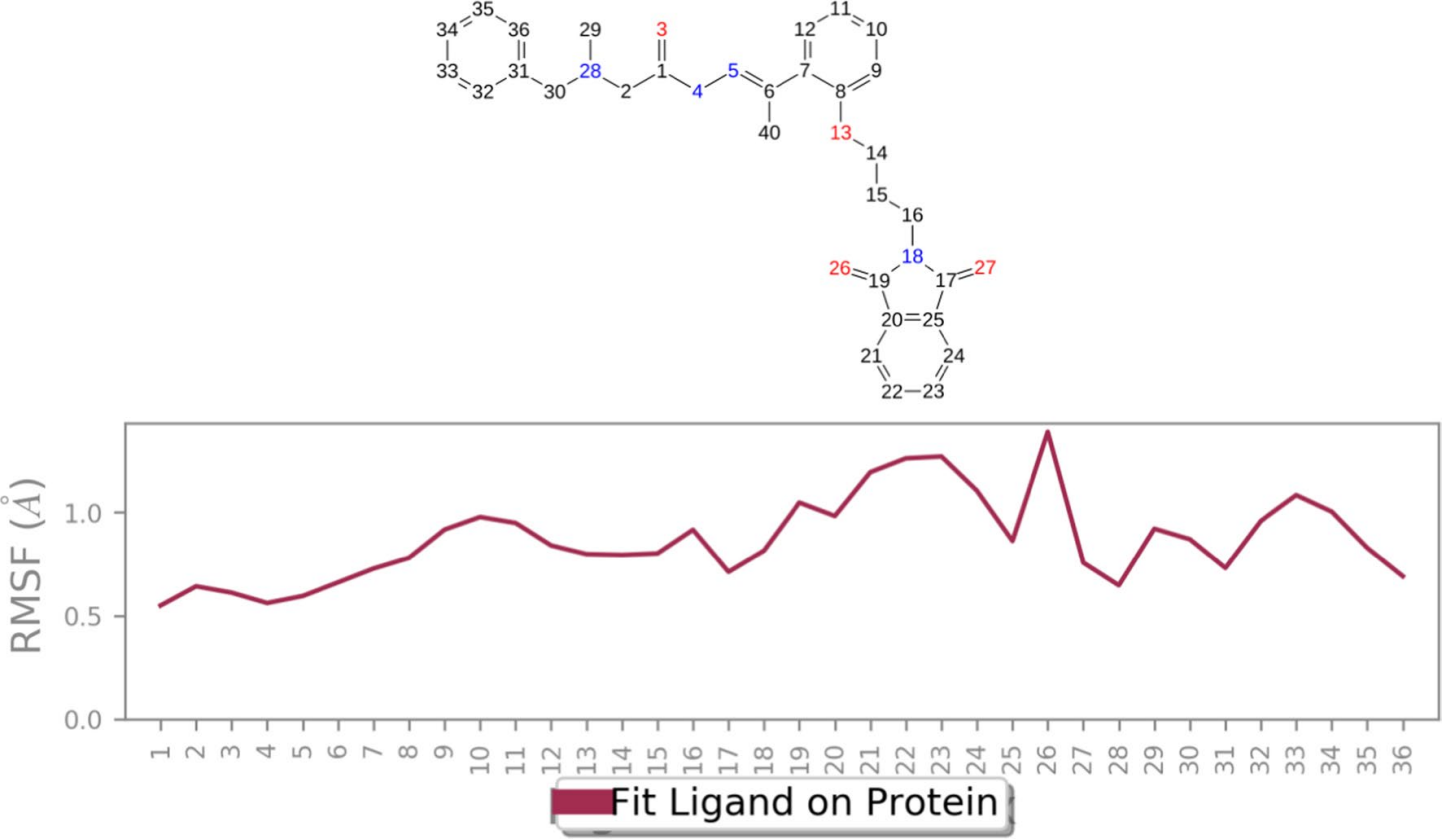
RMSF graph of the heavy atoms of **8a** in complex with AChE. The structure of **8a** is depicted, exhibiting the regions of the molecule with the highest fluctuations

Figure 10 illustrates RMSF values of the heavy atoms bound to AChE. Notably, all atoms constituting **8a** consistently exhibit RMSF values below 2 Å. This observation underscores the high stability observed within the complex formed between compound **8a** and AChE, primarily attributable to the presence of robust intermolecular interactions. These interactions play a crucial role in limiting the movement of the involved atoms throughout the molecular dynamics simulation, further validating the stability and integrity of the compound **8a**-AChE complex.

**Fig. 11:**
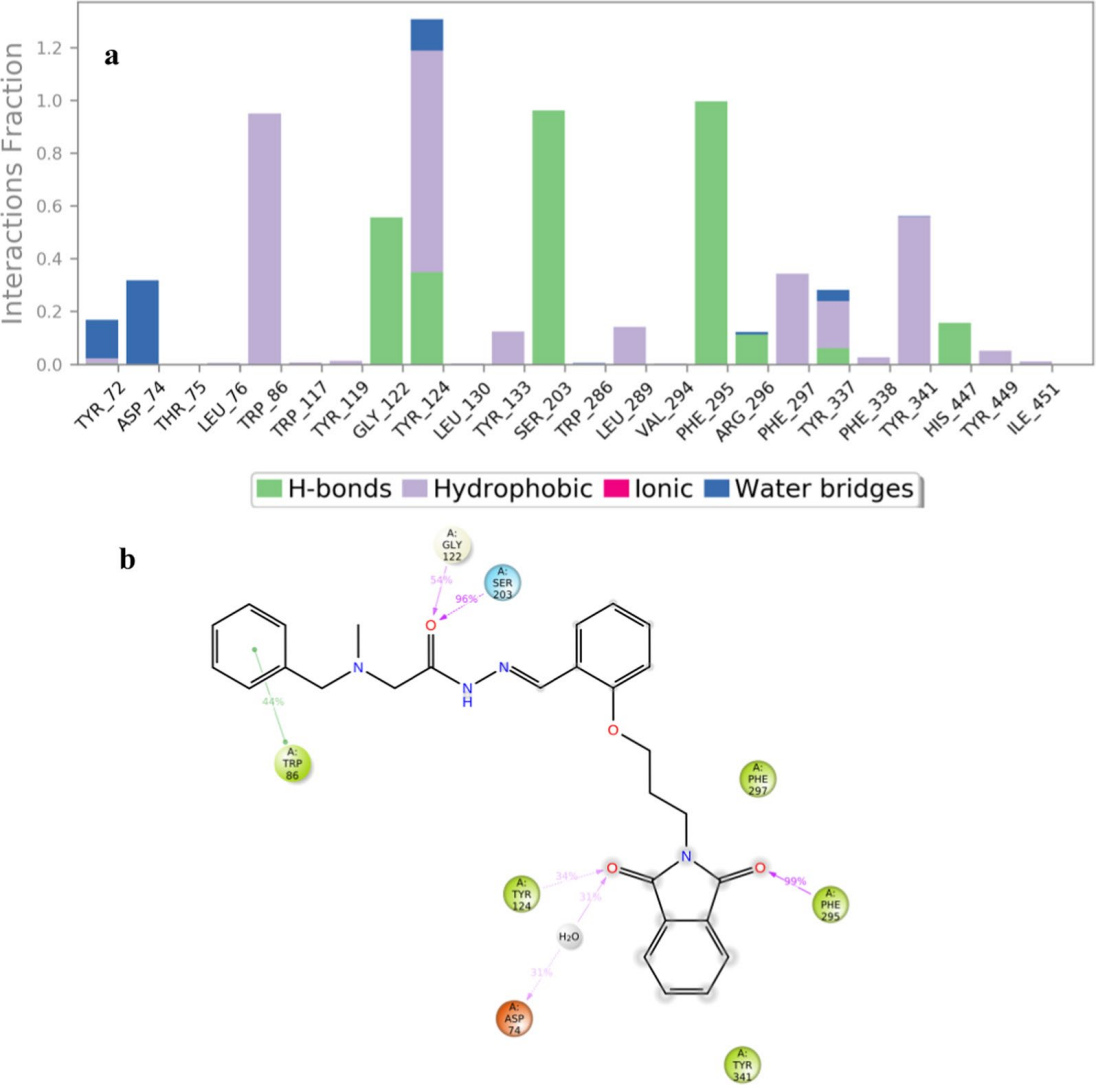
**a** Protein**-8a** contacts based on the type of interactions and **b** Protein residues interactions with **8a** atoms. H-Bound displayed in purple color

Next, a comprehensive evaluation of the diverse interactions between compound **8a** and AChE was conducted. As illustrated in Fig. 11a, hydrogen bonding interactions were prominently observed. Notably, Tyr124 exhibited 100% occupancy throughout the simulation, along with Phe295, Ser203, and Trp86, which consistently participated in interactions over the entire simulation period. These interactions played a pivotal role in anchoring the compound within the active site, aligning with its pronounced potency.

Furthermore, the 2D interactions diagram of compound **8a** in complex with the enzyme, derived from the analysis of MD trajectories, is depicted in Fig. 11b. The C=O group of isoindolin-1,3-dione engaged in a hydrogen-bonding interaction with Phe295 (~ 100%), while another C=O formed hydrogen bonds with Tyr124 (~ 34%) and participated in an additional hydrogen bond with Asp74 mediated by water (~ 33%). The C=O linker of the hydrazide demonstrated two hydrogen-bonding interactions with Gly222 and Ser203, contributing significantly to the ligand’s binding stability. Additionally, the terminal benzyl moiety exhibited pi-pi stacking interactions with Trp86 (~ 44%) in the PAS pocket, further enhancing the overall binding affinity and stability of the compound within the active site.

### Prime/MM-GBSA analysis

In addition to the interaction analysis, the Prime/MM-GBSA module was utilized to quantitatively assess the interaction strengths within the inhibitor/AChE complexes [25–27]. The computed ΔG_bind_ values for the complexes formed between compound **8a** and AChE were determined to be − 90.38 kcal/mol. This analysis provides valuable insights into the thermodynamic aspects of the binding interactions, further supporting the robust affinity and stability of the compound **8a**-AChE complexes.

### In silico drug-likeness, ADME, and toxicity studies

**Fig. 12:**
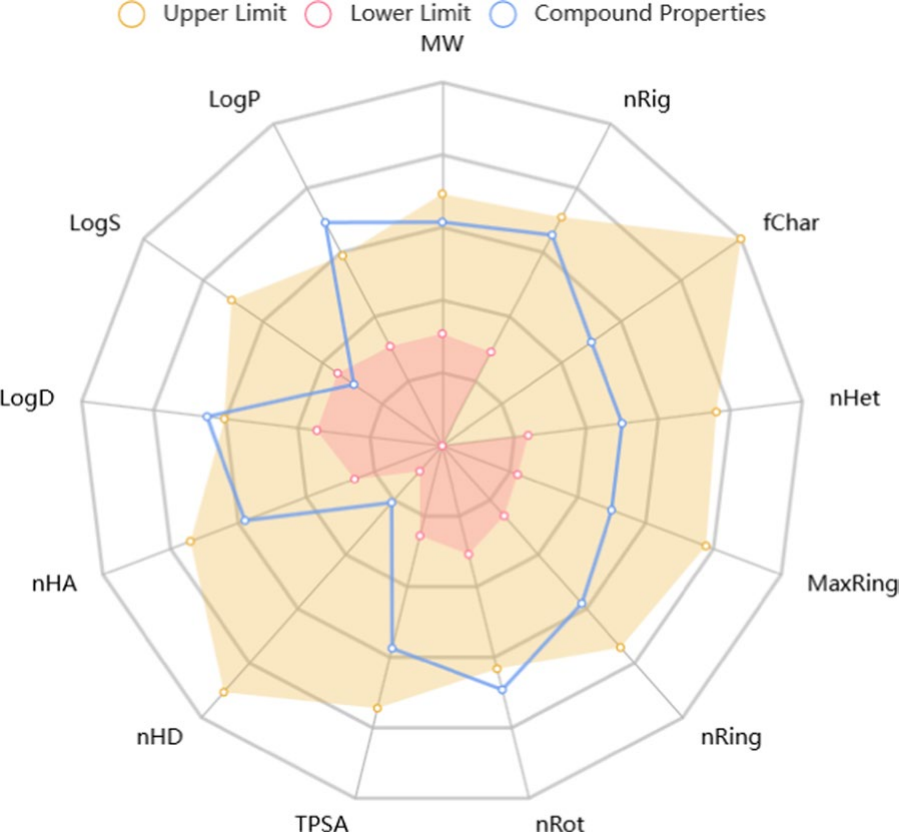
ADMET radar calculation of compound **8a**

The drug-likeness and ADME-T (absorption, distribution, metabolism, excretion, and toxicity) properties of the most potent compounds within the **8a** series were computationally assessed to estimate their potential as orally administered agents. The in silico predictions in Table 3 and Fig. 12 revealed that compound **8a** falls within the desirable range for properties defining an ideal oral therapeutic agent. Notably, it meets criteria such as bioavailability, stability, non-toxicity, and blood–brain barrier (BBB) penetration, encapsulating drug-like characteristics. These findings suggest that compound 8a holds promise as a candidate for further development as an orally administered therapeutic agent.

**Table 3:** Physicochemical properties of compound **8a**

	Parameters	Value		Parameters	Value
Physicochemical property	Molecular weight	498.23	Distribution	VD	0.551
	Volume	520.78		BBB penetration	+
	Density	0.957	Metabolism	CYP1A2 inhibitor	–
	nHA	8		CYP1A2 substrate	+
	nHD	1		CYP2C9 inhibitor	+
	nRot	13		CYP2C9 substrate	+
	nRing	4		CYP2D6 inhibitor	+
	MaxRing	9		CYP2D6 substrate	+
	nHet	8	Excretion	CL	5.826
	fChar	0		T1/2	0.659
	TPSA	91.31	Toxicity	AMES toxicity	–
	logP	4.094		H-HT	–
	logD	3.377		AMES toxicity	–
Absorption	Caco-2 permeability	− 5.135		Carcinogencity	–
	MDCK permeability	3.2e−05		Skin sensitization	–
	Pgp-inhibitor	–		Acute toxicity rule	0
	Pgp-substrate	–	Medicinal chemistry	SAscore	2.325
	HLA	+		Lipinski rule	Accepted

## Conclusion

In summary, this study presents the design and synthesis of a novel series of acetohydrazide derivatives based on isoindolin-1,3-dione (compounds **8a**–**h**). The synthesized compounds were evaluated for their inhibitory activity against both AChE and BChE. Notably, the synthesized compounds exhibited remarkable potency against AChE, with IC_50_ values ranging from 0.11 ± 0.05 to 0.86 ± 0.02 µM, whereas their activity against BChE was in the range of 5.7 ± 0.2 to 30.2 ± 2.8 µM. Among the tested compounds, **8a** emerged as the most potent inhibitor against AChE, and the kinetic analysis revealed that compound **8a** functions as a competitive inhibitor, with a *K*_*i*_ value of 0.0886 µM. The binding interactions of the most potent compound (**8a**) with the active site of AChE were elucidated through molecular docking and molecular dynamic simulations. These results underscore the significant interactions formed by the potent compound within the AChE active site.

Molecular dynamics simulations further supported the favorable binding state of compound **8a** in the AChE active site. Additionally*, *in silico pharmacokinetic studies predicted satisfactory pharmacokinetics for the promising compound **8a**, suggesting its viability as a potential drug candidate. Specifically, the oral administration of compound **8a** is anticipated to result in favorable pharmacokinetic profiles, reinforcing its potential as a promising therapeutic agent.

## Experimental

### General methods

All the chemicals were purchased from Merck, Germany, and Sigma, Germany. Melting points of compounds **8a**–**h** were measured with a Kofler hot stage apparatus. IR spectra of these compounds were recorded with a Nicolet Magna FTIR 550 spectrophotometer (KBr disks). ^1^H NMR spectra were recorded using a Varian spectrometer 500 MHz instrument using DMSO-*d*_*6*_ as solvent.^13^C NMR spectra were obtained at 125 MHz and referenced to the internal solvent signals. Chemical shifts were reported in parts per million (ppm). Multiplicities were indicated by s (singlet), d (doublet), t (triplet), q (quartet), m (multiplet), coupling constant J was reported in hertz (Hz).

### General synthesis procedure of (benzyl-alkyl-amino)-acetic acid ethyl esters (3)

To a solution of K_2_CO_3_ (10 mmol) and secondary amines **1a** or **1b** (5 mmol) in DMF (20 mL) was added ethyl bromoacetate (7.5 mmol). The reaction mixture was stirred at room temperature for 16 h and the reaction progress was monitored by Thin-Layer Chromatography (TLC) utilizing *n*-hexane:ethyl acetate (1:2). After completion reaction, the reaction mixture was diluted with ethyl acetate (20 mL), washed twice with water (20 mL) to remove DMF, and washed with brine. The collected organic layers were extracted and dried over Na_2_SO_4_, filtered, and concentrated to afford the product as colorless oil in high yields (80–95%).

#### General synthesis procedure of (benzyl-N-alkyl-amino)-acetic acid hydrazide (4)

Compound (**3a** or **3b**) (50 mmol) was dissolved in ethanol (50 ml), and hydrazine hydrate (100 mmol) was added to the solution and the mixture was refluxed for onr hour. An additional amount of hydrazine hydrate (60 mmol) was added and the reaction mixture was refluxed for an additional 4 h. The reaction was monitored by TLC, and after completion, the liqiud was extracted by CH_2_Cl_2_ and dried over Na_2_SO_4_, decanted, and concentrated to afford the product as colorless oil in good yields (Additional file 1).

#### General synthesis procedure of (3-(1,3-dioxoisoindolin-2-yl)propoxy)benzaldehyde 7a–d

A mixture of phthalimide **5** (1 mmol), hydroxybezaldehyde derivative **6** (1 mmol), and K_2_CO_3_ (1.3 mmol) in DMF (10 mL) was heated at 80 °C for 8 h. Upon completion of the following reaction, the mixture was poured into crushed ice, and the precipitate was filtered off and dried, affording aldehyde **7** [28].

#### General synthesis procedure of (benzyl-N-alkyl amino)-acetic acid 4-[3-(1,3-dioxo-1,3-dihydro-isoindol-2-yl)-propoxy]-benzylidene-hydrazide (8a–h)

To a solution of hydrazide **4a** or **4b** (0.01 mol) and catalytic amount of p-TSA, were added substituted aldehydes **7a**–**d** (0.01 mol). The reaction mixture was refluxed for the appropriate time. The reaction is being monitored by TLC using hexane: ethyl acetate (4:6). After completion of the reaction, the solid product was filtered, washed with ethanol and, dried under reduced pressure, and recrystallized in ethanol to yield the pure compound.

##### 2-(benzyl(methyl)amino)-Nʹ-(2-(3-(1,3-dioxoisoindolin-2-yl)propoxy)benzylidene)acetohydrazide (8a)

White solid, yield = 78%; Mp: 142–145 °C. Elemental analysis (%) calcd for C_28_H_28_N_4_O_4_ (M = 484.21): C, 69.41; H, 5.82; N, 11.56; found: C, 69.40; H, 5.72 N, 11.6; ^1^H NMR (500MHz, CDCl_3_): δ = 2.25–2.30 (m, 2H, –OCH_2_–C***H***_2_), 2.41 (s, 3H, C***H***_3_–N), 3.26 (s, 2H, C***H***_2_–CO), 3.69 (s, 2H, Ph-C***H***_2_–N), 3.97 (dd, *J* = 6.5,7Hz, C***H***_2_–NCO), 4.11 (dd, *J* = 5.5, 6Hz, C***H***_2_–O–Ph), 6.87 (d, 1H, *J* = 8Hz, H3ʹ), 6.96 (t, 1H, *J* = 7.5Hz, H5ʹ), 7.23–7.35 (m, 5H, H2,3,4,5,6), 7.41 (m, 1H, H4ʹ), 7.68–7.69 (m, 2H, Hb, Hc), 7.77–7.79 (m, 2H, Ha, Hd), 8.0 (d, 1H, *J* = 7.5Hz, H6ʹ), 8.56 (s, 1H, C***H***=N), 10.1 (s, N***H***); ^13^C NMR (125 MHz, CDCl_3_) δ = 28.41, 35.09, 35.22, 42.54, 43.55, 60.05, 62.52, 65.77, 66.20, 76.85, 77.10, 77.36, 112.02, 112.50, 121.18, 121.22, 122.28, 123.23, 123.32, 126.05, 127.21, 127.62, 128.31, 128.61, 129.10, 129.37, 131.37, 131.69, 131.96, 132.02, 134.06, 134.13, 137.62, 143.73, 157.13, 166.66, 168.33.

##### 2-(benzyl(ethyl)amino)-Nʹ-(2-(3-(1,3-dioxoisoindolin-2-yl)propoxy)benzylidene)acetohydrazide (8b)

White solid, yield = 76%;Mp: 159–160 °C. Elemental analysis (%) calcd for C_30_H_32_N_4_O_5_ (M = 528.24): C, 68.17; H, 6.10; N, 10.60; found: C, 68.16; H, 6.09 N, 10.61; ^1^H NMR (500MHz, CDCl_3_): δ = 1.22 (t, 3H, J = 7.0Hz, C***H***_3_), 2.29 (m, 2H, –OCH_2_–C***H***_2_–), 2.79 (m, 2H, CH_3_–C***H***_2_–N), 3.32 (s, 2H, C***H***_2_–CO), 3.75 (s, 2H, Ph–C***H***_2_–N), 3.99 (dd, J = 6.5, 7Hz, C***H***_2_–NCO), 4.12 (dd, J = 5.5, 6Hz, C***H***_2_–O–Ph), 6.89 (d, 1H, *J* = 8Hz, H3ʹ), 6.97 (t, 1H, J = 7.5Hz, H5ʹ), 7.23–7.44 (m, 6H, H2,3,4,5,6, H4ʹ), 7.71 (m, 2H, Hb, Hc), 7.81 (m, 2H, Ha, Hd), 8.08 (d, 1H, J = 7.5Hz, H6ʹ), 8.5 (s, 1H, C***H***=N), 10.2 (s, N***H***); ^13^CNMR (125 MHz, CDCl_3_): 12.1, 28.4, 34.9,40.6, 49.2, 59.1, 66.6, 111.9, 113.0, 121.1, 122.3, 127.2, 127.3, 127.5, 128.6, 129.0, 131.6, 132.0, 134.0, 143.6, 157.0, 168.3.

##### 2-(benzyl(ethyl)amino)-Nʹ-(2-(3-(1,3-dioxoisoindolin-2-yl)propoxy)-5-nitrobenzylidene)acetohydrazide (8c)

Yellowsolid, yield = 82%;Mp: 164–166 °C. Elemental analysis (%) calcd for C_29_H_29_N_5_O_6_ (M = 543.21): C, 64.08; H, 5.38; N, 12.88; found: C, 64.06; H, 5.41 N, 12.69;^1^H NMR (500MHz, CDCl_3_): δ = 1.20 (t, 3H, J = 7.5Hz, C***H***_3_), 2.29–2.34 (m, 2H, –OCH_2_–C***H***_2_–), 2.70–2.74 (m, 2H,CH_3_–C***H***_2_–N), 3.34 (s, 2H, C***H***_2_–CO), 3.72 (s, 2H, Ph–C***H***_2_–N), 3.97 (dd, 2H, J = 6.5, 7Hz, C***H***_2_–NCO), 4.22 (dd, 2H, J = 5.5,6Hz, C***H***_2_–O–Ph), 6.95 (d, 1H, J = 8Hz, H3ʹ), 7.23 (dd, 1H, J = 7, 7.5Hz, H4) 7.31–7.34 (m, 2H, H3,5), 7.40 (d, 2H, J = 8Hz, H2,6), 7.69–7.70 (m, 2H, Hb, Hc), 7.77–7.79 (m, 2H, Ha, Hd), 8.16–8.19 (m, 1H, H4ʹ), 8.59 (s, 1H, H6ʹ), 8.83 (s, 1H,C***H***=N), 10.3 (s, N***H***). ^13^CNMR (125 MHz, CDCl_3_): δ = 12.1, 28.1, 34.6, 49.3, 56.9, 58.0, 59.2, 66.4, 111.7, 122.7, 123.3, 126.7, 127.6, 128.2, 128.6, 129.0, 129.1, 131.9, 134.2, 134.3, 137.8, 141.6, 141.8, 161.0, 167.7, 168.3

##### 2-(benzyl(ethyl)amino)-N'-(3-(3-(1,3-dioxoisoindolin-2-yl)propoxy)benzylidene)acetohydrazide (8d)

White solid, yield = 75%;Mp: 167–169 °C; Chemical Formula: C_29_H_30_N_4_O_4_. Elemental analysis (%) calcd for C_29_H_30_N_4_O_4_ (M = 498.23): C, 69.86; H, 6.07; N, 11.24; found: C, 69.88; H, 6.08; N, 11.20;^1^H NMR (500 MHz, cdcl_3_) δ 1.16 (t, 3H, J = 7.0Hz, C***H***_**3**_), 2.18–2.21 (m, 2H, –OCH_2_–C***H***_2_–), 2.66–2.70 (m, 2H, CH_3_–C***H***_2_–N), 3.28 (s, 2H, C***H***_2_–CO), 3.70 (s, 2H, Ph–C***H***_2_–N), 3.92 (t, 2H, J = 6.5Hz, C***H***_2_–NCO), 4.07 (t, 2H, J = 5.5Hz, C***H***_2_–O–Ph), 6.85 (m, 1H, H6ʹ), 7.25–7.37 (m, 8H, H2,3,4,5,6, H2ʹ, H4ʹ, H5ʹ), 7.72 (m, 2H,Hb, Hc), 7.84 (m, 2H, Ha, Hd), 8.02 (s, 1H, C***H***=N), 10.1 (s, N***H***); ^13^C NMR (125 MHz, CDCl_3_): δ = 12.20, 28.25, 35.43, 49.32, 57.03, 59.27, 65.72, 76.84, 77.10, 77.35, 112.12, 117.64, 120.98, 123.25, 127.68, 128.25, 128.72, 128.95, 129.57, 132.16, 133.93, 134.90, 137.79, 148.19, 159.01, 167.39, 168.34.

##### 2-(benzyl(ethyl)amino)-Nʹ-(3-(3-(1,3-dioxoisoindolin-2-yl)propoxy)-4-methoxybenzylidene)acetohydrazide (8e)

Creamsolid, yield = 76%;Mp: 164–166 °C; Chemical Formula: C_30_H_32_N_4_O_5;_Elemental analysis (%) calcd for C_30_H_32_N_4_O_5_ (M = 528.24): C, 68.17; H, 6.10; N, 10.60; found: C, 68.18; H, 6.11; N, 10.58;^1^H NMR (500MHz, CDCl_3_): δ = 1.19 (t, 3H, J = 7.0Hz, C***H***_**3**_), 2.19–2.22 (m, 2H, –OCH_2_–C***H***_2_–), 2.69–2.74 (m, 2H, CH_3_–C***H***_2_–N), 3.34 (s, 2H, C***H***_2_–CO), 3.74 (s, 2H, Ph–C***H***_2_–N), 3.86 (s, 3H, OC***H***_3_), 4.01 (dd, 2H, J = 6.5, 7Hz, C***H***_2_–NCO), 4.10 (dd, 2H, J = 5.5, 6Hz, C***H***_2_–O–Ph), 6.94 (d, 1H, J = 8Hz, H6ʹ), 7.06 (dd, 1H, J = 8, 4Hz, H4), 7.23 (dd, 1H, J = 8, 4Hz, H5ʹ)7.5Hz), 7.28–7.31 (m, 3H, H2,6, H3ʹ), 7.38 (m, 2H, H3, 5), 7.70–7.74 (m, 2H, Hb, Hc), 7.79–7.83 (m, 2H, Ha, Hd), 8.6 (s, 1H, C***H***=N), 10.4 (s, N***H***);^13^CNMR (125 MHz, CDCl_3_): δ = 12.0, 29.4, 35.3, 49.0, 55.8, 57.0, 59.2, 70.9, 114.0, 118.7, 123.2, 123.5, 124.0, 127.4, 127.5, 128.5, 128.9, 132.0, 134.0, 134.3, 144.0, 152.3, 168.4

##### 2-(benzyl(methyl)amino)-Nʹ-(4-(3-(1,3-dioxoisoindolin-2-yl)propoxy)benzylidene)acetohydrazide (8f)

White solid, yield = 78%;Mp: 163–164 °C; Elemental analysis (%) calcd for C_28_H_28_N_4_O_4_ (M = 484.21): C, 69.41; H, 5.82; N, 11.56; found: C, 69.40; H, 5.82; N, 11.58; ^1^H NMR (500MHz, CDCl_3_): δ = 2.19–2.24 (m, 2H, C***H***_2_–CH_2_–O), 2.41 (s, 3H, C***H***_3_–N), 3.27 (s, 2H, NCO–C***H***_*2*_–NCH_3_), 3.69 (s, 2H, Ph–C***H***_2_–N), 3.93 (dd, 2H, J = 6.5, 7Hz, C***H***_2_–NCO), 4.08 (t, 2H, J = 6Hz, C***H***_*2*_–O), 6.79 (d, 2H, J = 8.5Hz, H2ʹ, 6ʹ), 7.31–7.42 (m, 5H, H2, 3, 4, 5, 6), 7.65 (d, J = 8.5Hz, 2H, H3ʹ, H5ʹ), 7.73–7.74 (m, 2H, Hb, Hc), 7.84–7.86 (m, 2H, Ha, Hd), 8.10 (s, 1H, C***H***=N), 10.08 (s, NH); ^13^C NMR (125 MHz, CDCl_3_): δ = 28.2, 35.4, 43.3, 59.8, 62.4, 65.8, 76.7, 77.0, 77.2, 114.5, 114.6, 123.2, 123.3, 126.2, 127.8, 128.5, 128.6, 129.0, 129.3, 132.1, 133.9, 148.1, 160.7, 168.3

##### 2-(benzyl(ethyl)amino)-Nʹ-(4-(3-(1,3-dioxoisoindolin-2-yl)propoxy)benzylidene)acetohydrazide (8g)

White solid, yield = 78%; Mp: 174–176 °C; Elemental analysis (%) calcd for C_29_H_30_N_4_O_4_ (M = 498.23): C, 69.86; H, 6.07; N, 11.24; found: C, 69.88; H, 6.09; N, 11.22;^1^H NMR (500MHz, CDCl3): δ = 1.17 (t, 3H, J = 7.0Hz, CH_3_), 2.19–2.24 (m, 2H, –OCH_2_–C***H***_2_–), 2.69 (m, 2H, CH_3_–C***H***_2_–N), 3.29 (s, 2H, C***H***_2_–CO), 3.71 (s, 2H, Ph–C***H***_2_–N), 3.93 (dd, 2H, J = 6.5, 7Hz, C**H**_2_–NCO), 4.08 (dd, 2H, J = 5.5, 6Hz, C***H***_**2**_–O–Ph), 6.80 (d, 2H, J = 8Hz, H2ʹ, 6ʹ), 7.28–7.39 (m, 5H, H2, 3, 4, 5, 6), 7.65 (d, J = 8Hz, 2H, H3ʹ, H5ʹ), 7.73–7.74 (m, 2H, Hb, Hc), 7.84–7.86 (m, 2H, Ha, Hd), 8.03 (s, 1H, C***H***=N), 10.0 (s, N***H***); ^13^CNMR (125 MHz, CDCl_3_): δ = 12.1, 28.2, 35.4, 49.2, 59.2, 65.8, 114.5, 123.2, 126.2, 127.6, 128.7, 128.9, 129.2, 132.1, 133.9, 147.9, 160.6, 168.3

##### 2-(benzyl(ethyl)amino)-Nʹ-(4-(3-(1,3-dioxoisoindolin-2-yl)propoxy)-3-methoxybenzylidene)acetohydrazide (8h)

White solid, yield = 77%;Mp: 164–166 °C; Elemental analysis (%) calcd for C_30_H_32_N_4_O_5_ (M = 528.24): C, 68.17; H, 6.10; N, 10.60; found: C, 68.18; H, 6.09; N, 10.60; ^1^H NMR (500MHz, CDCl_3_): δ = 1.15 (t, 3H, J = 7.0Hz, C***H***_3_–N), 2.23–2.29 (m, 2H, C***H***_2_–CH_2_–O), 2.65–2.69 (m, 2H, CH_3_–C***H***_2_–N), 3.26 (s, 2H, N–C***H***_2_–CO), 3.68 (s, 5H, C***H***_3_O, Ph–C***H***_**2**_–N), 3.92 (dd, J = 6.5, 7Hz, C***H***_2_–NCO), 4.08 (t, J = 6Hz, C***H***_2_–O–Ph), 6.82 (d, 1H, J = 8Hz, H6ʹ), 7.06 (d, 1H, J = 8Hz, H5ʹ), 7.27–7.37 (m, 6H, H2, 3, 4, 5, 6, H3ʹ), 7.70–7.71 (m, 2H, Hb, Hc), 7.81–7.83 (m, 2H, Ha, Hd), 8.00 (s, 1H, C***H***=N), 10.0 (s, N***H***); ^13^C NMR (125 MHz, CDCl_3_): δ = 168.34, 150.59, 149.69, 148.38, 133.84, 132.23, 128.93, 128.69, 127.65, 126.68, 123.15, 122.77, 112.00, 108.59, 66.95, 59.24, 56.98, 55.86, 49.26, 35.64, 28.29, 12.19.

### Enzyme inhibition assays

The inhibitory potency of derivatives were determined using a modified Ellman method. Briefly, various concentrations of the inhibitors were added to 200 μL sodium phosphate buffer (0.1 mol/L, pH 7.4), 20 µL AChE or BChE and 20 µL DTNB (301 μM) in separate wells of a 96-well microplate, gently mixed, and incubated at 37 °C for 15 min. Acetylthiocholine (ATCh) or butyrylthiocholine (BTCh) (20 μL, final concentration of 452 μM) was then added to generate the yellow anion of 5-thio-2-nitrobenzoic acid. The absorbance of each well was measured at 415 nm using a microplate reader. IC50 values were calculated with GraphPad Prism software, representing the mean of three independent experiments and expressed as mean ± SEM [29].

### Enzyme kinetics

The ChE inhibition reactions were determined by Ellman’s assay at different concentrations of **8a** and **8g** [30]. Lineweaver–Burk reciprocal plots (1/v vs. 1/[s]) were constructed at varying concentrations of the substrate acetylthiocholine (0.1–1 mM) to obtain the type of inhibition. The inhibition constant *K*_*i*_ was calculated by the plot of slopes *versus* the corresponding concentrations of the inhibitors.

### Docking study

To explore the interactions of compounds **8a** and **8g** within the binding pockets of AChE (PDBID: 4EY7) and BChE (PDBID: 4BDS), a docking study was conducted using the Schrödinger package LLC (Maestro, Schrödinger, LLC, New York, NY, 2021). The proteins were prepared and optimized via the protein preparation wizard by removing water molecules and cognate ligands from the receptors. Hydrogen atoms were added, and non-polar hydrogens were merged into related atoms of the receptors during the protein preparation process and finally, minimization was executed by the OPLS4 force field.

For ligand preparation, the 2D structures of the ligands were drawn in ChemDraw version 12, converted into SDF files, and subjected to the LigPrep module. Ligands were then prepared using the OPLS2005 force field with the EPIK method.The docking procedure involved induced-fit docking with a box size of 20 Å, and 10 poses per ligand were generated to form the final complex. This comprehensive approach aimed to elucidate the potential binding modes and interactions of compounds **8a** and **8g** with the active sites of AChE and BChE.

### Molecular dynamic simulation

In this study, the molecular dynamics simulation was carried out using the Desmond v5.3 module, which is integrated into the Maestro interface from Schrodinger. The induced fit docking (IFD) method was used to obtain the appropriate pose for the molecular dynamic simulation procedure of the compounds. In order to conduct molecular dynamic simulation, the first step involved solvating the protein–ligand complexes with explicit SPC water molecules and positioning them at the center of an appropriately sized orthorhombic box under Periodic Boundary Conditions. To mimic real cellular ionic concentrations, counterions and a 0.15 M solution of NaCl were added to neutralize the system. The molecular dynamic protocol consisted of three steps: minimization, pre-production, and production MD simulations. The system was allowed to relax for 2500 steps by the steepest descent approach to minimize the energy. Next, a small force constant was applied to the enzyme as the system's temperature gradually rose from 0 to 300 K to prevent abrupt changes. MD simulations were executed under the NPT (constant number of atoms, constant pressure: 1.01325 bar, and constant temperature: 300 K) ensemble, utilizing the Nose–Hoover chain method as the default. Long-range electrostatic forces were calculated using the Particle-mesh-based Ewald approach, with a cutoff radius for Columbia forces set to 9.0 Å. The protein–ligand complex underwent 100 ns of production molecular dynamic simulations, with data frames stored every 1000 ps during the simulation.

### Free binding energy calculations

Binding free energy calculation of protein–ligand complex was performed by using mechanic/poisson-boltzmann surface area (MM/PBSA) methodbased on the following equation

$$\Delta G_{Bind} = E_{Complex} - \left( {E_{Receptor} + E_{Ligand} } \right)$$

where ΔG_Bind_ is the calculated relative free energy in which it includes both receptor and ligand strain energy. E_Complex_ is defined as the MM-GBSA energy of the minimized complex, and E_Ligand_is the MM-GBSA energy of the ligand after removing it from the complex and allowing it to relax. E_Receptor_ is the MM-GBSA energy of relaxed protein after separating it from the ligand.

### Prediction of pharmacokinetic properties of synthesis compounds

The physicochemical and biological absorption, distribution, metabolism, excretion, and toxicity properties of the selected compound were performed using the ADMETlab 2.0 server (https://admetmesh.scbdd.com/) (accessed on: 12-9-2023).

### Supplementary Information

**Additional file 1: Fig. S1.** ^1^H-NMR and ^13^C-NMR of **8a**. **Fig. S2.** ^1^H-NMR and ^13^C-NMR of **8b**. **Fig. S3.** ^1^H-NMR and ^13^C-NMR of **8c**. **Fig. S4.** ^1^H-NMR and ^13^C-NMR of **8d**. **Fig. S5.** ^1^H-NMR and ^13^C-NMR of **8e**. **Fig. S6.** ^1^H-NMR and ^13^C-NMR of **8f**. **Fig. S7.** ^1^H-NMR and ^13^C-NMR of **8g**. **Fig. S8.** ^1^H-NMR and ^13^C-NMR of **8h**.

## Data Availability

All data generated or analysed during this study are included in this published article and its additional information file.
