# Supplementary material for: Anticholinesterase activities of novel isoindolin-1,3-dione-based acetohydrazide derivatives: design, synthesis, biological evaluation, molecular dynamic study
Source: BMC Chem. 2024 Apr 1;18(1):64. doi: 10.1186/s13065-024-01169-4 (PMC10985906; doi:10.1186/s13065-024-01169-4)
Supplement: Supplementary file 1 — Additional file 1: Fig. S1. 1H-NMR and 13C-NMR of 8a. Fig. S2. 1H-NMR and 13C-NMR of 8b. Fig. S3. 1H-NMR and 13C-NMR of 8c. Fig. S4. 1H-NMR and 13C-NMR of 8d. Fig. S5. 1H-NMR and 13C-NMR of 8e. Fig. S6. 1H-NMR and 13C-NMR of 8f. Fig. S7. 1H-NMR and 13C-NMR of 8g. Fig. S8. 1H-NMR and 13C-NMR of 8h. [file 13065_2024_1169_MOESM1_ESM.pdf]

Fig. S1. ^{1}H-NMR and ^{13}C-NMR of **8a**

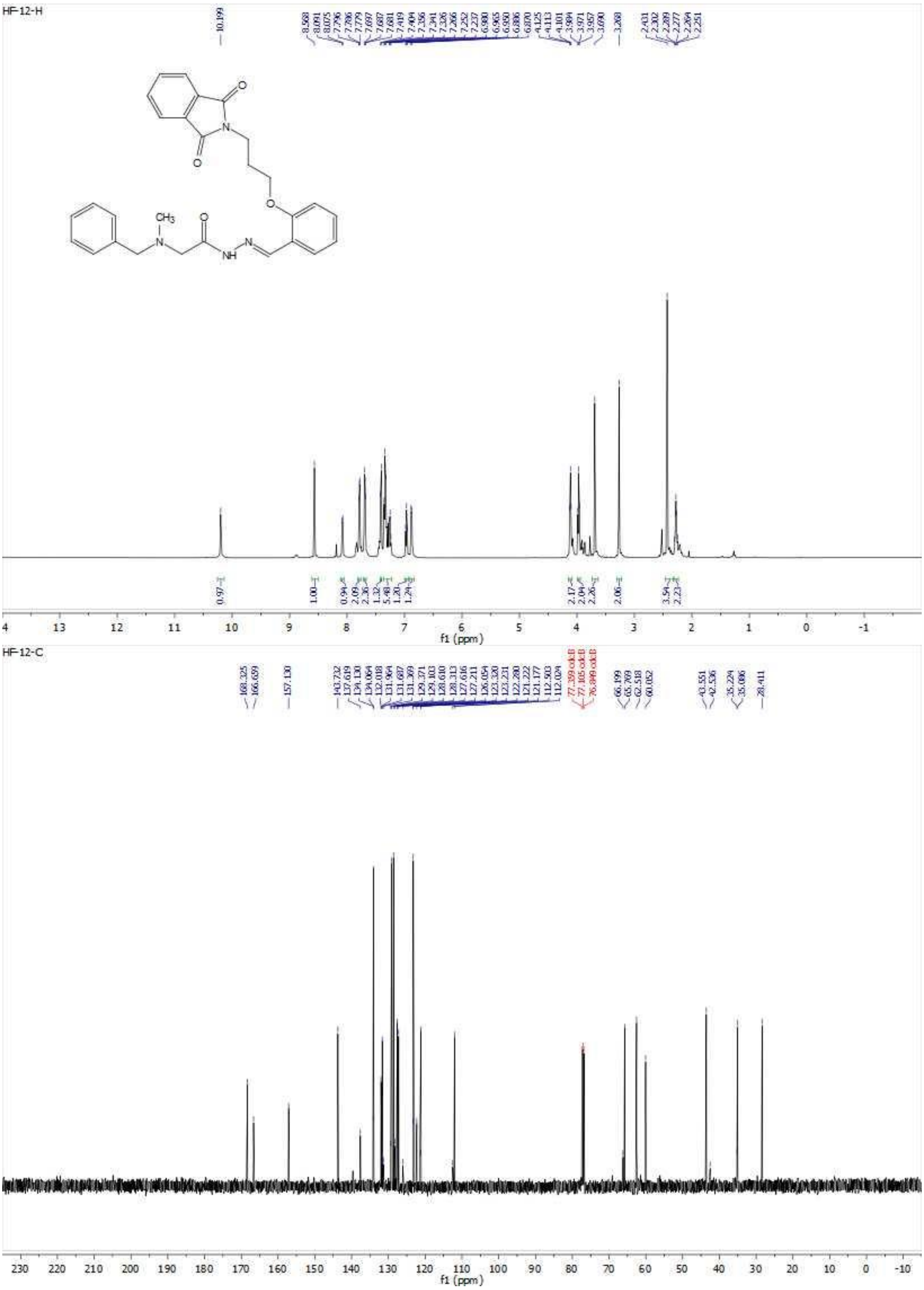

Fig. S2. ^{1}H-NMR and ^{13}C-NMr of **8b**

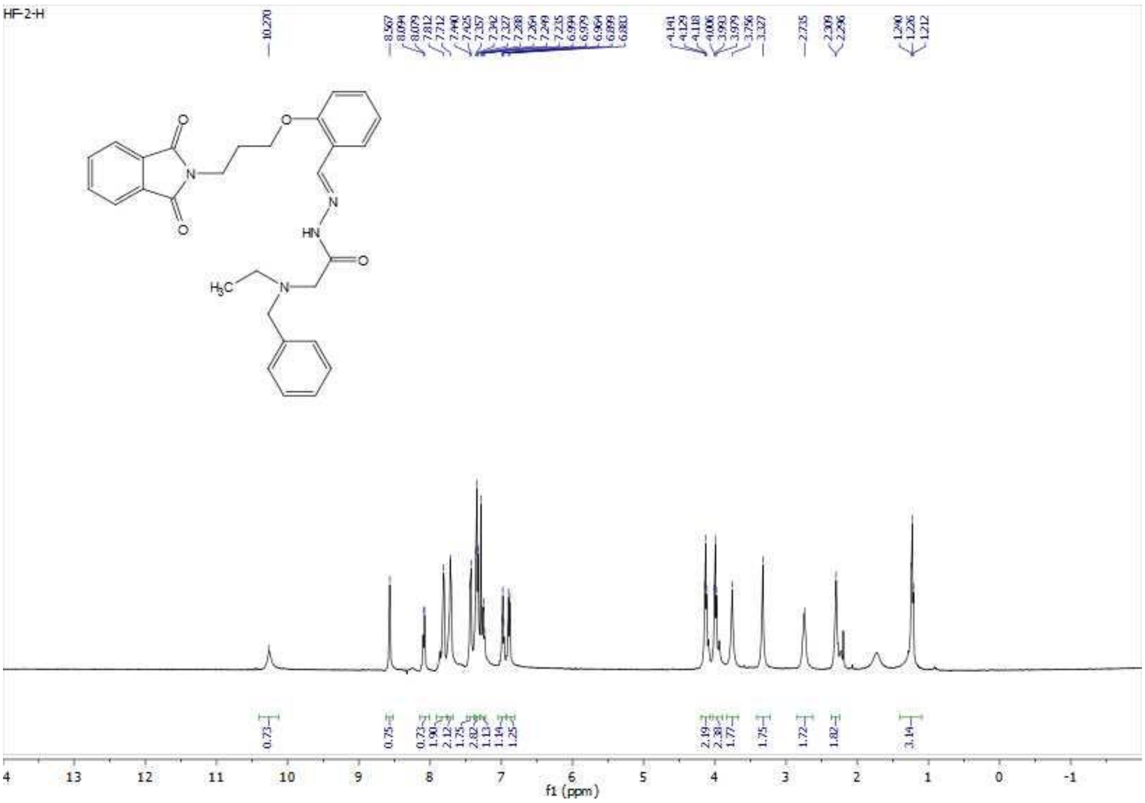

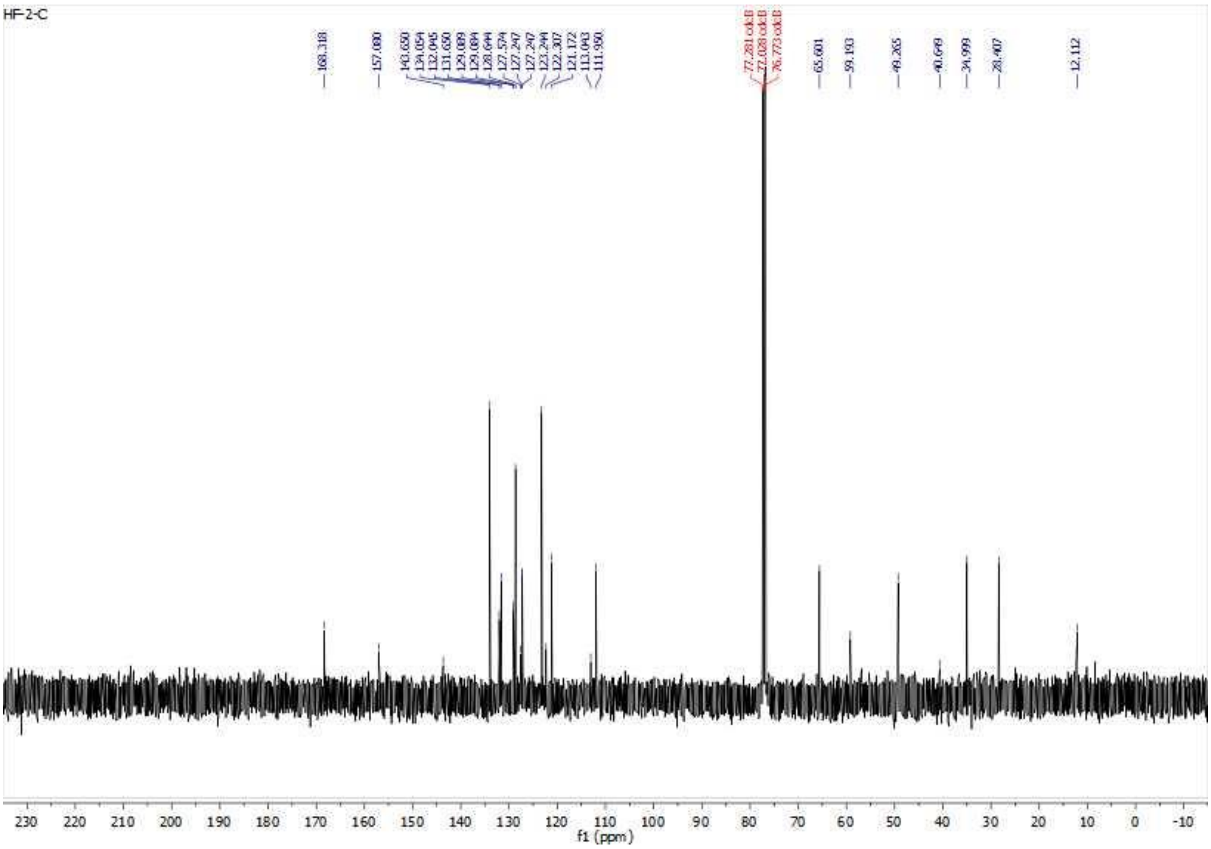

Fig. S3. ^{1}H-NMR and ^{13}C-NMR of **8c**

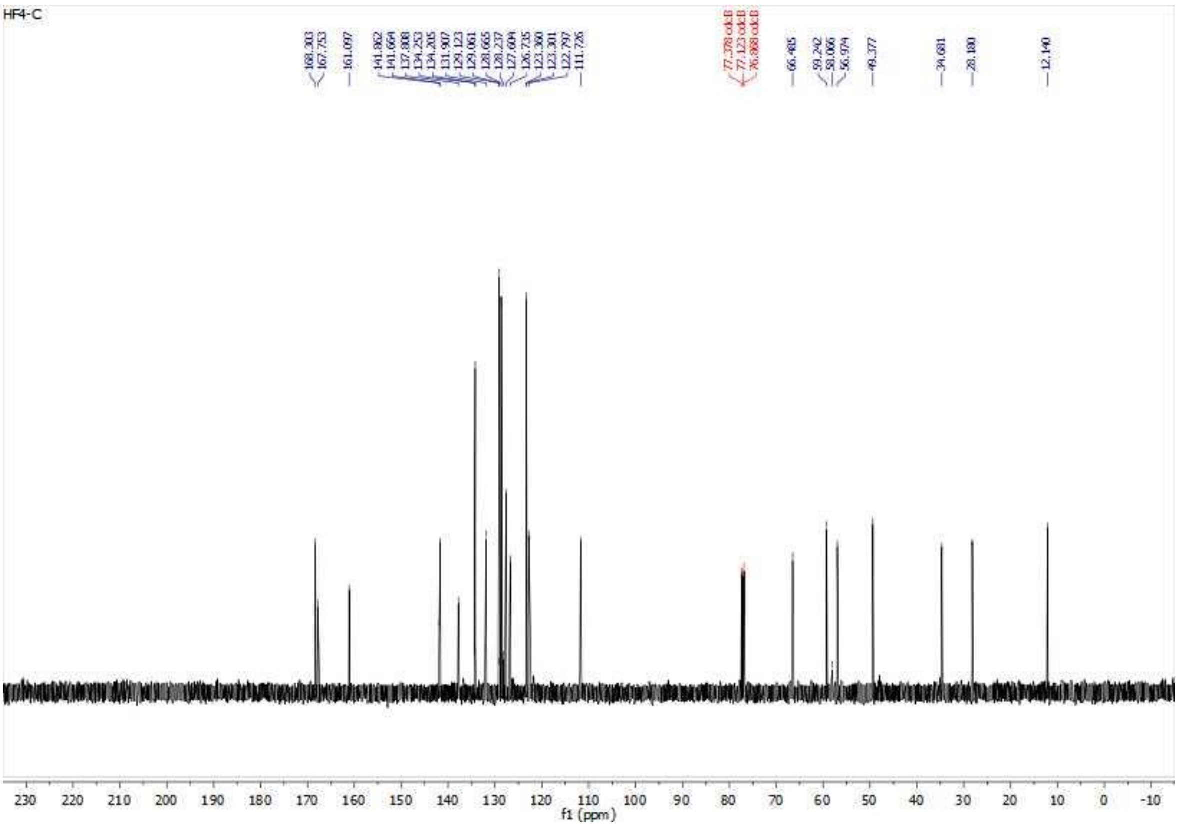

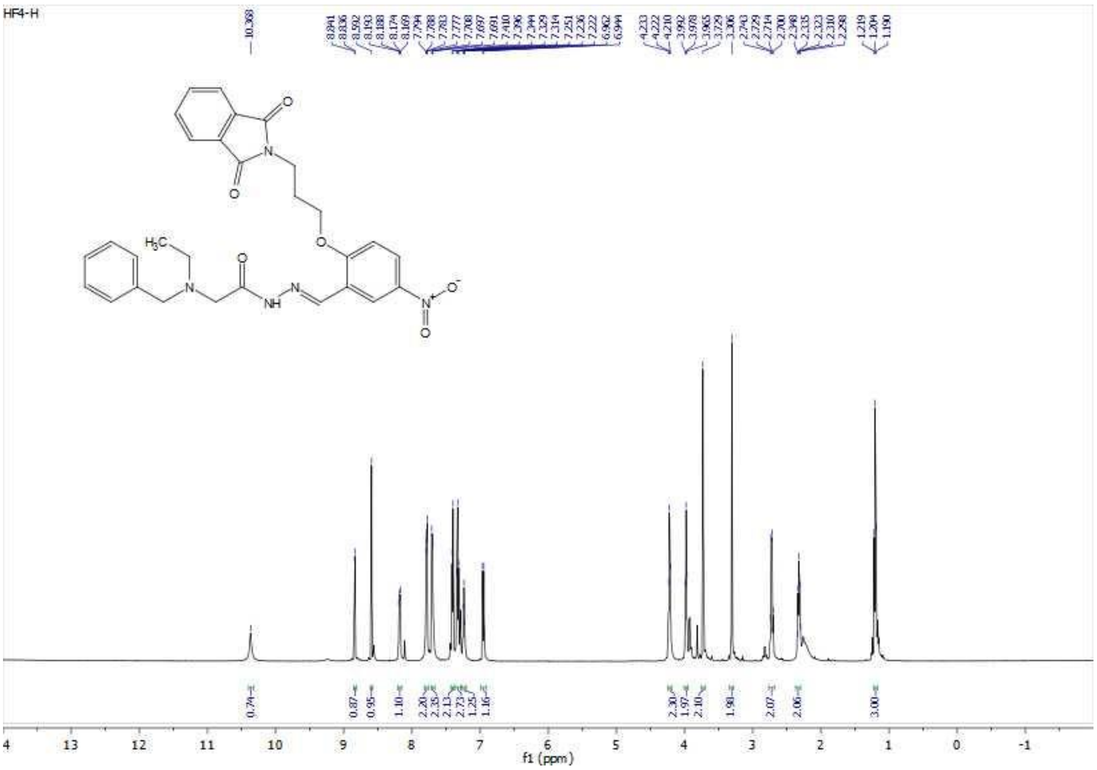

Fig. S4. ^{1}H-NMR and ^{13}C-NMR of **8d**

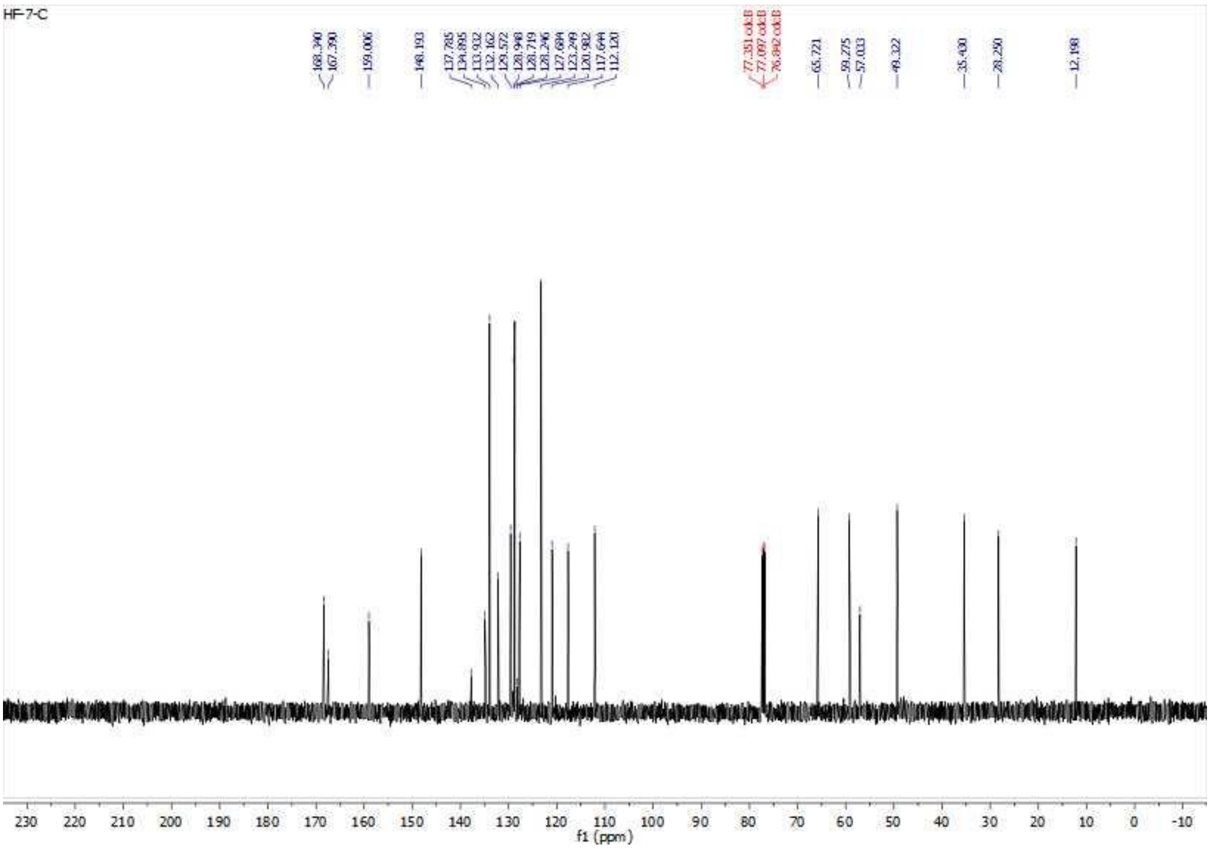

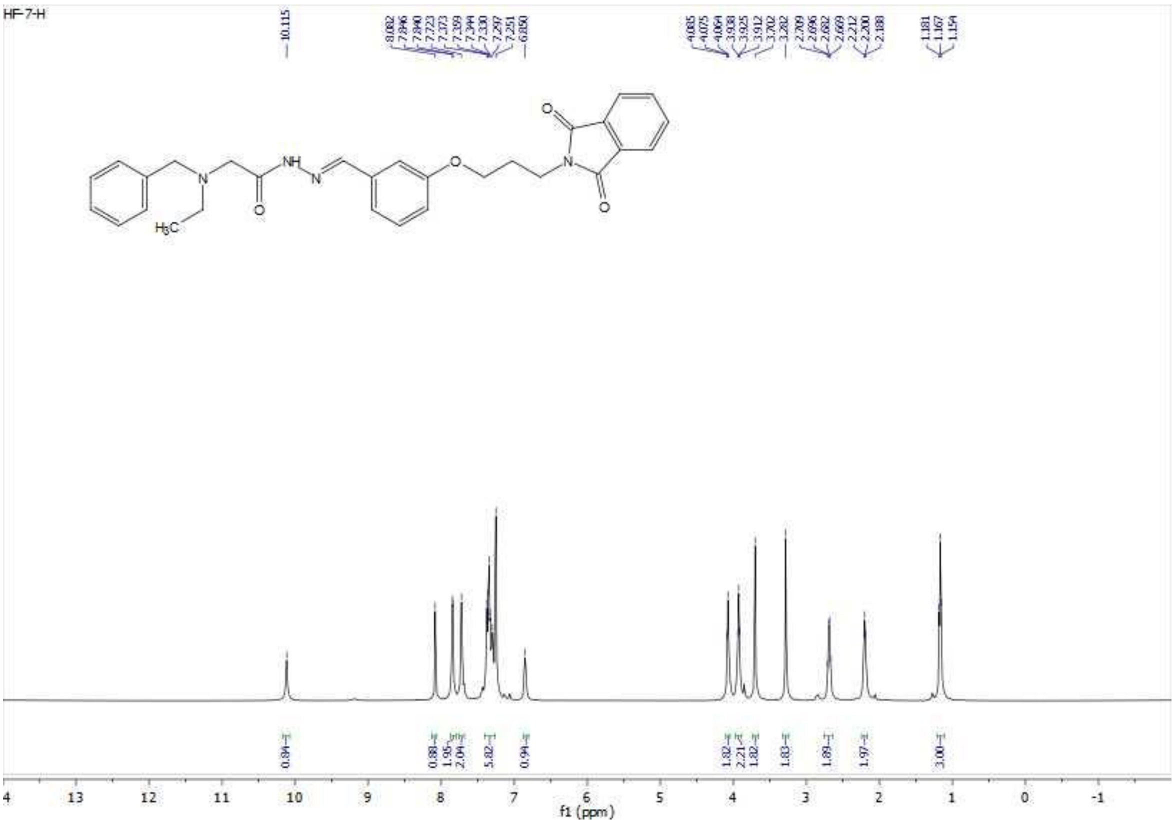

Fig. S5. ^{1}H-NMR and ^{13}C-NMr of **8e**

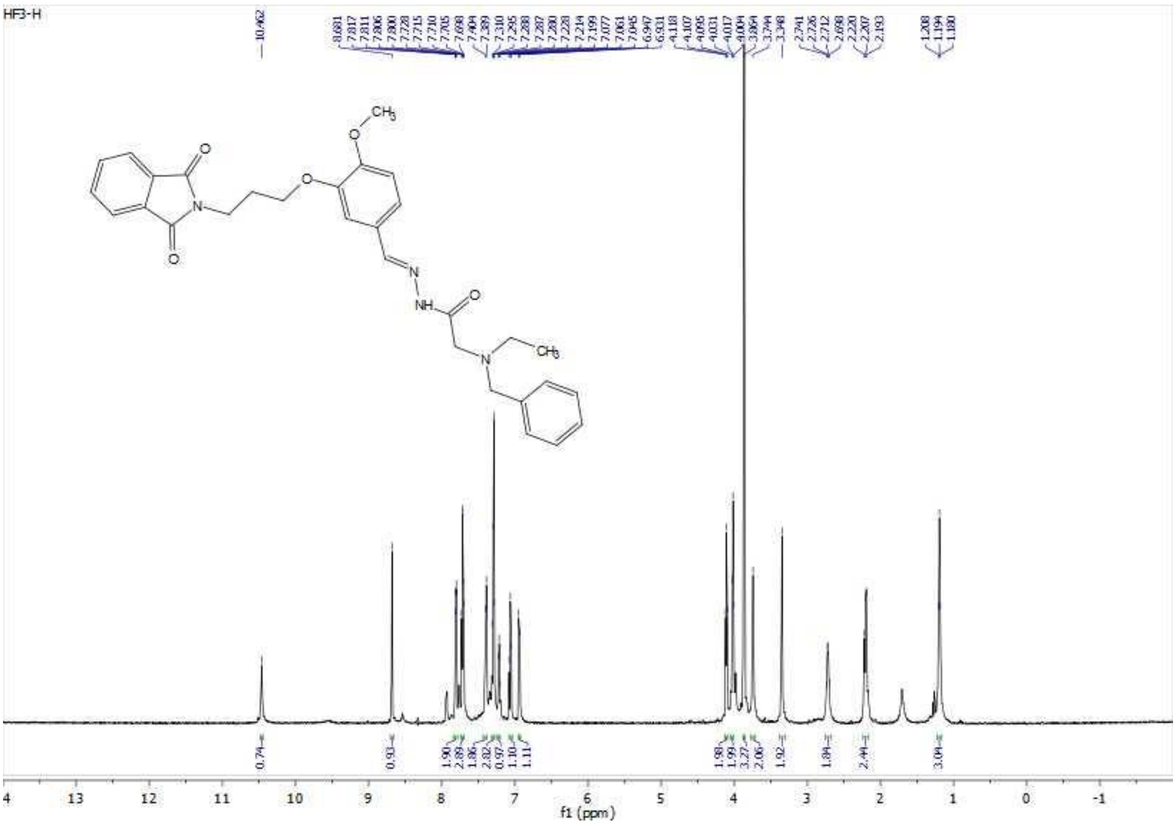

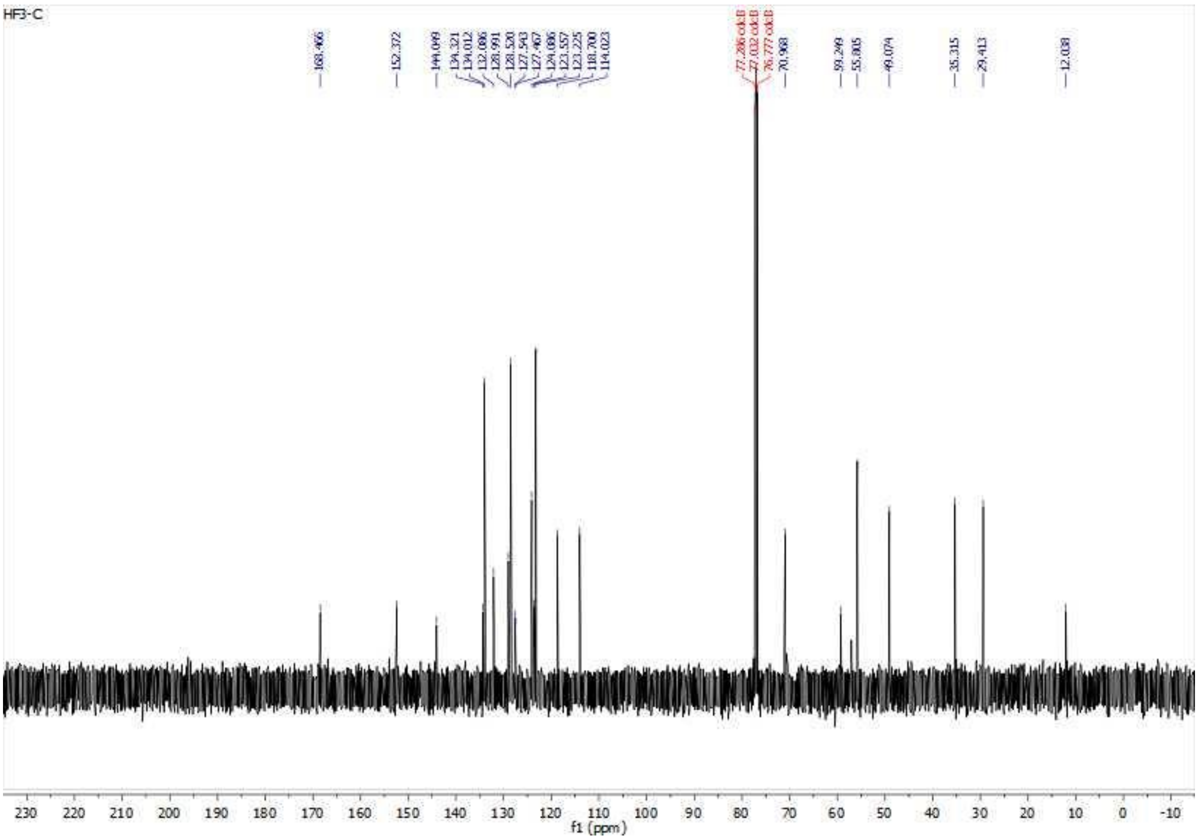

Fig. S6. ^{1}H-NMR and ^{13}C-NMR of **8f**

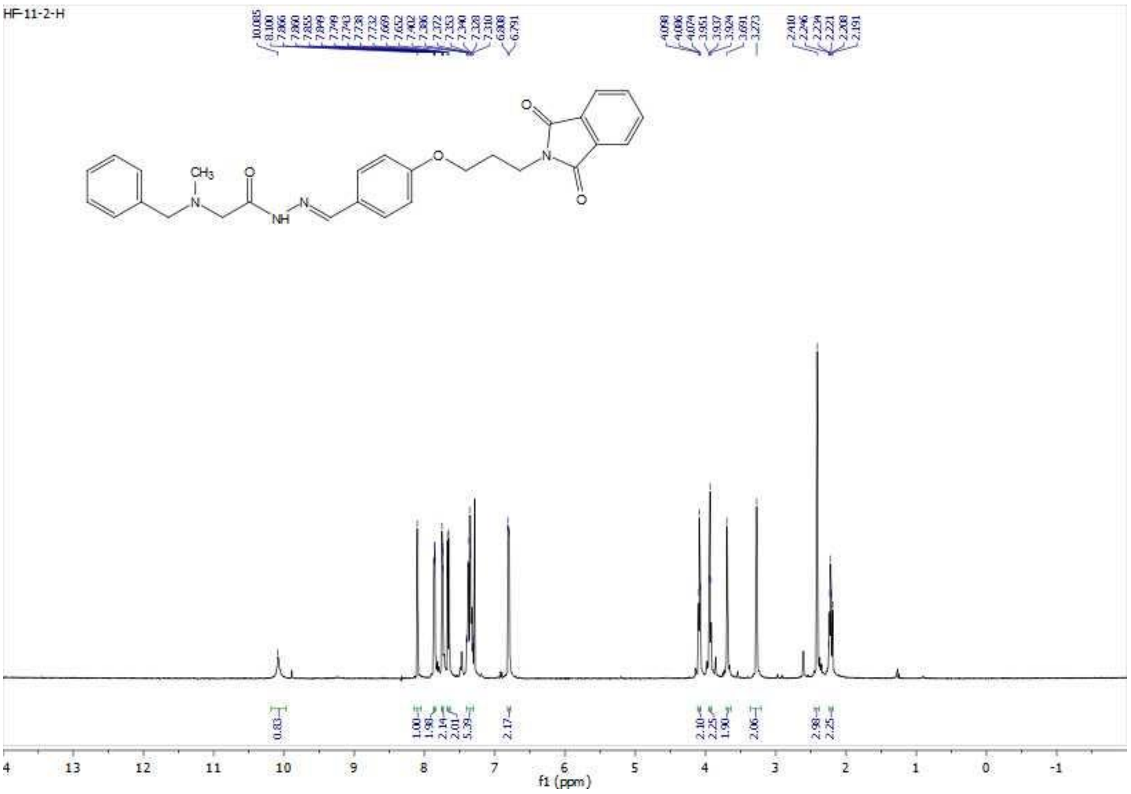

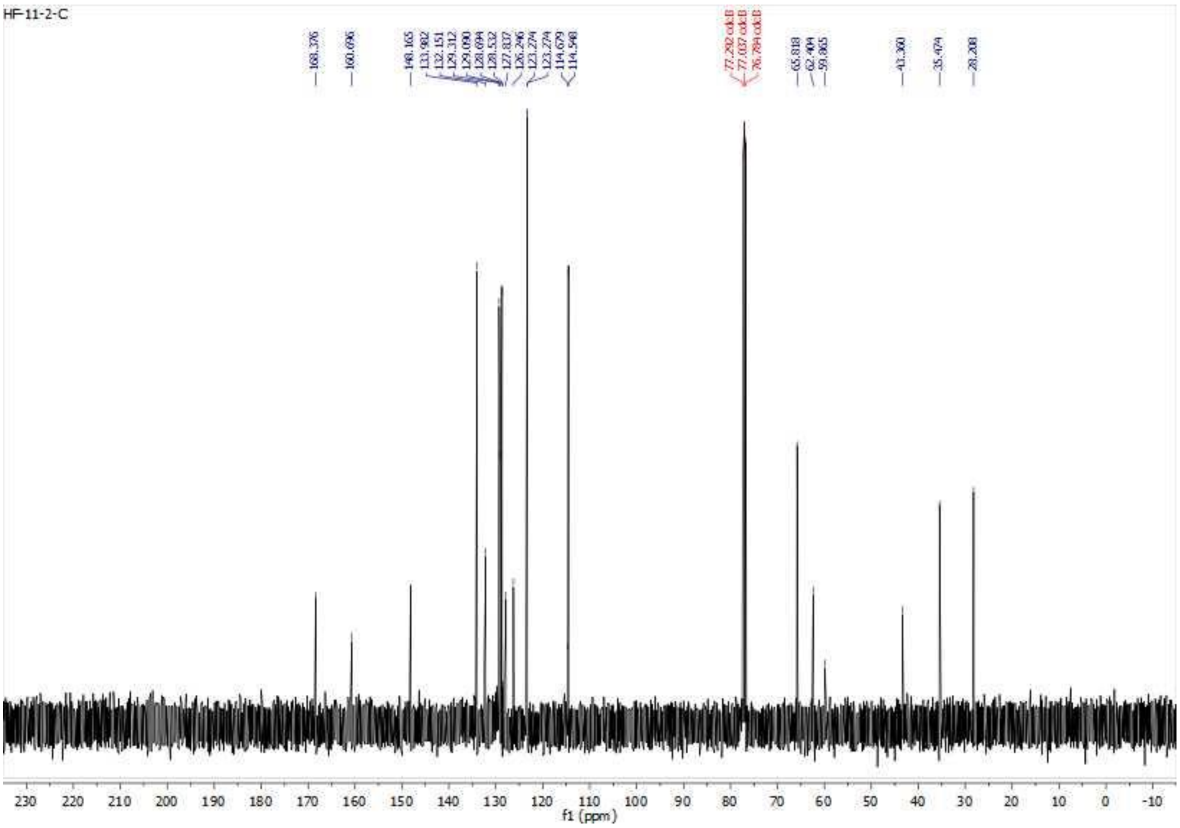

Fig. S7. ^{1}H-NMR and ^{13}C-NMr of **8g**

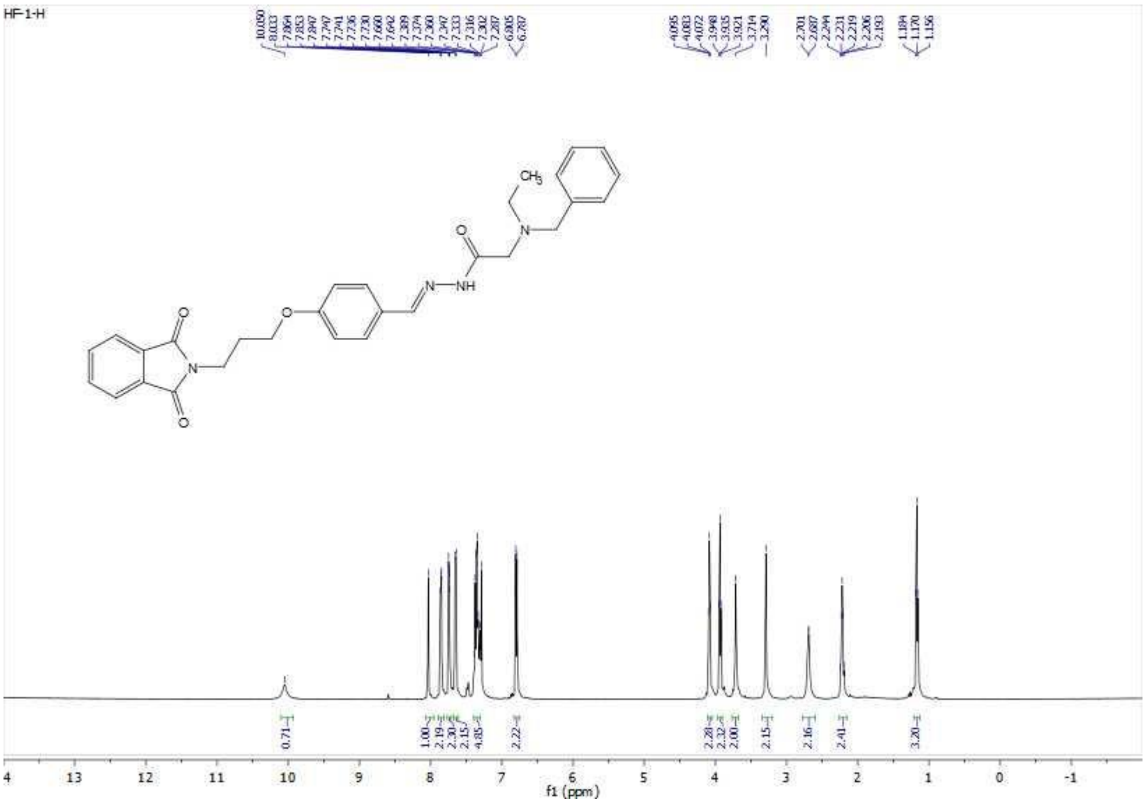

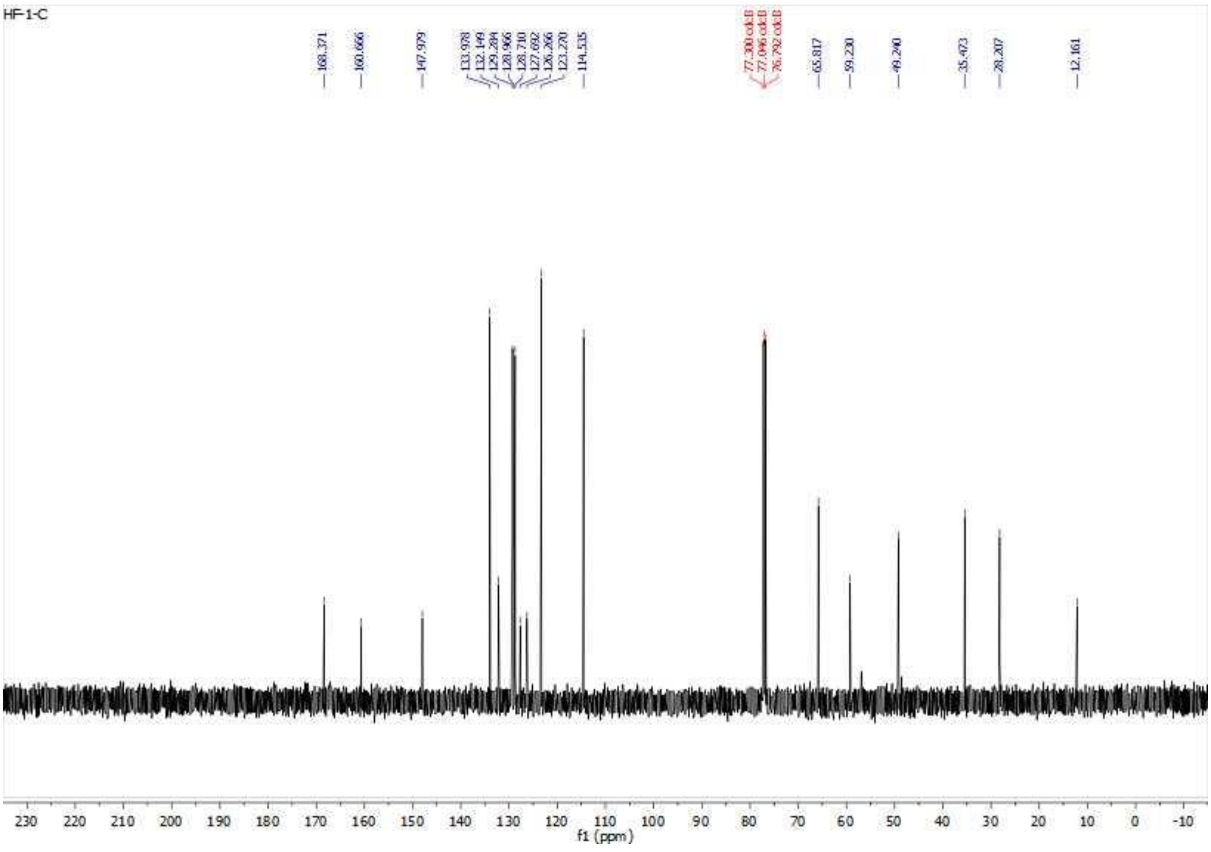

Fig. S8. ^{1}H-NMR and ^{13}C-NMR of **8h**

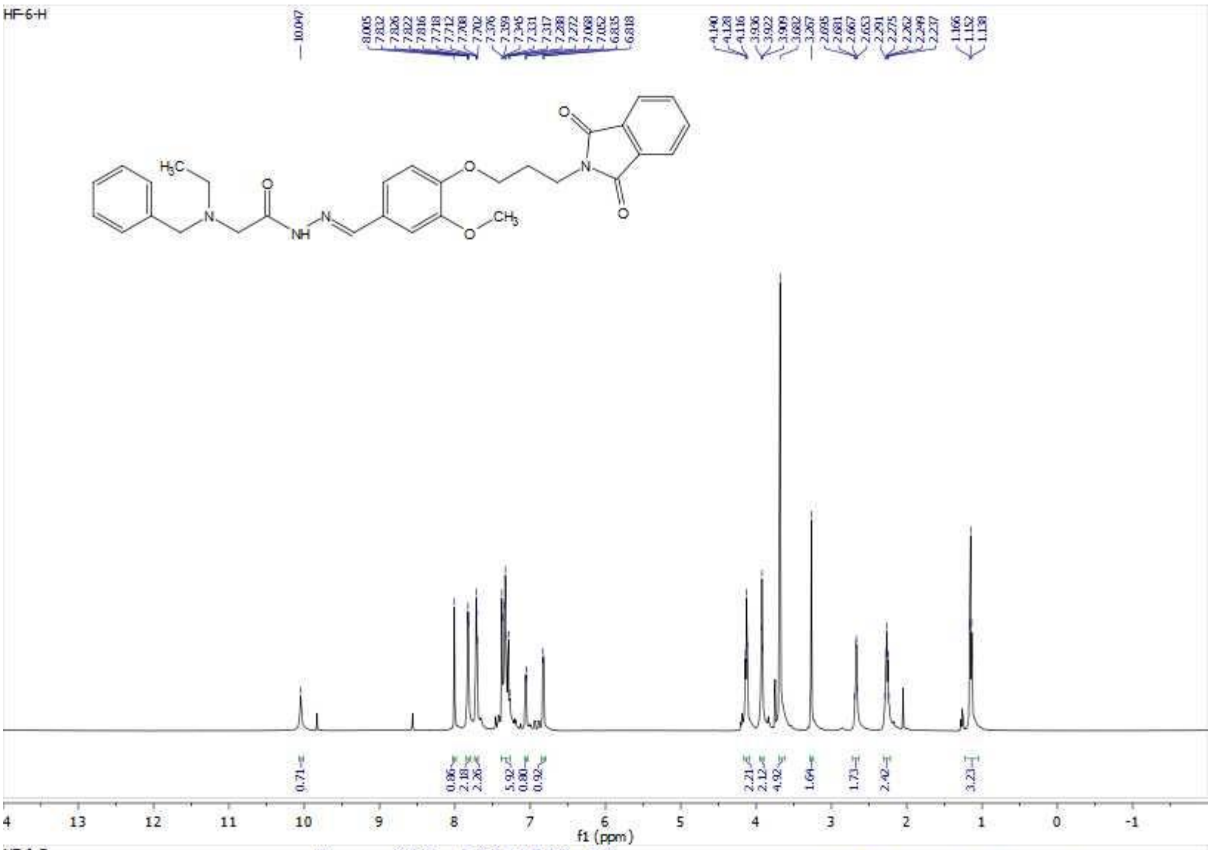
HF-6-H
10.047
8.005 7.832 7.826 7.822 7.816 7.718 7.712 7.708 7.702 7.376 7.359 7.345 7.331 7.317 7.288 7.272 7.068 7.052 6.835 6.818
4.140 4.128 4.116 3.936 3.922 3.909 3.682 3.267 2.695 2.681 2.667 2.653 2.291 2.275 2.262 2.249 2.237 1.166 1.152 1.138
0.71 0.86 2.18 2.26 5.92 0.80 0.92 2.21 2.12 4.92 1.64 1.73 2.42 3.21
f1 (ppm)

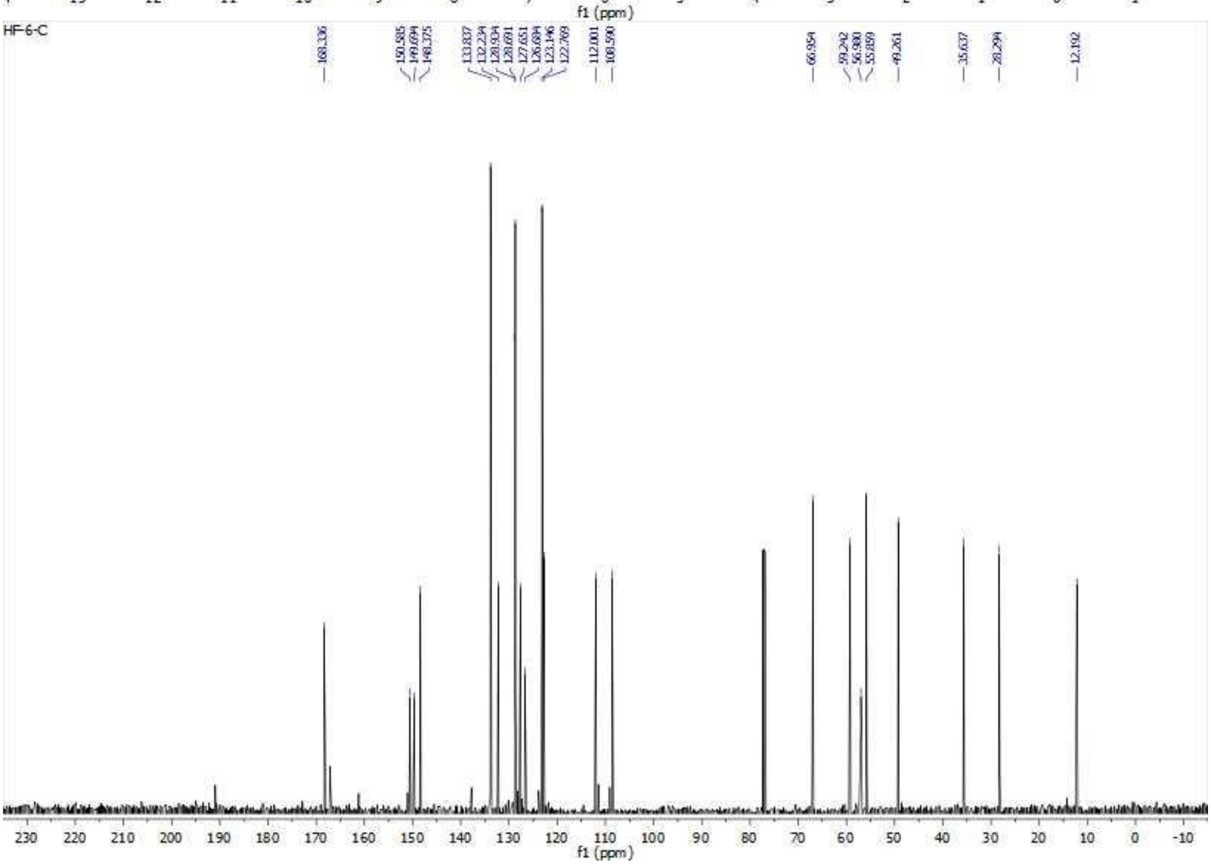
HF-6-C
168.336
150.585 149.694 148.375
133.837 132.234 128.934 128.691 127.651 126.694 123.146 122.769
112.001 108.590
66.954 59.242 56.980 55.859 49.261 35.637 28.294 12.192
f1 (ppm)
